# Blood and urinary cytokine balance and renal outcomes at cardiac surgery

**DOI:** 10.1186/s12882-021-02621-6

**Published:** 2021-12-08

**Authors:** William T. McBride, Mary Jo Kurth, Anna Domanska, Joanne Watt, Gavin McLean, Jijin Joseph, John V. Lamont, Peter Fitzgerald, Mark W. Ruddock

**Affiliations:** 1 Department of Cardiac Anesthesia, Belfast Health & Social Care Trust, 274 Grosvenor Road, Belfast, Northern Ireland BT12 6BA UK; 2 Randox Laboratories Ltd, Clinical Studies Group, 55 Diamond Road, Crumlin, County Antrim BT29 4QY Northern Ireland, UK

**Keywords:** Acute kidney injury, Biomarkers, Cytokines, Cardiac surgery, TNFsr2, IL-1RA, IL-12p40, H-FABP, Midkine

## Abstract

**Background:**

Increased perioperative pro-inflammatory biomarkers, renal hypoperfusion and ischemia reperfusion injury (IRI) heighten cardiac surgery acute kidney injury (CS-AKI) risk. Increased urinary anti-inflammatory cytokines attenuate risk. We evaluated whether blood and urinary anti-inflammatory biomarkers, when expressed as ratios with biomarkers of inflammation, hypoperfusion and IRI are increased in CS-AKI patients.

**Methods:**

Preoperative and 24-h postoperative blood and urinary pro-inflammatory and anti-inflammatory cytokines, blood VEGF and H-FABP (hypoperfusion biomarkers), and MK, a biomarker for IRI, were measured in 401 cardiac surgery patients. Pre- and postoperative concentrations of biomarkers and selected ratios thereof, were compared between non-CS-AKI and CS-AKI patients.

**Results:**

Compared with non-CS-AKI, blood pro-inflammatory (pre- and post-op TNFα, IP-10, IL-12p40, MIP-1α, NGAL; pre-op IL-6; post-op IL-8, MK) and anti-inflammatory (pre- and post-op sTNFsr1, sTNFsr2, IL-1RA) biomarkers together with urinary pro-inflammatory (pre- and post-op uIL-12p40; post-op uIP-10, uNGAL) and anti-inflammatory (pre- and post-op usTNFsr1, usTNFsr2, uIL-1RA) biomarkers, were significantly higher in CS-AKI patients. Urinary anti-inflammatory biomarkers, when expressed as ratios with biomarkers of inflammation (blood and urine), hypoperfusion (blood H-FABP and VEGF) and IRI (blood MK) were decreased in CS-AKI. In contrast, blood anti-inflammatory biomarkers expressed as similar ratios with blood biomarkers were increased in CS-AKI.

**Conclusions:**

The urinary anti-inflammatory response may protect against the injurious effects of perioperative inflammation, hypoperfusion and IRI. These finding may have clinical utility in bioprediction and earlier diagnosis of CS-AKI and informing future therapeutic strategies for CS-AKI patients.

**Supplementary Information:**

The online version contains supplementary material available at 10.1186/s12882-021-02621-6.

## Background

### Three major causes of CS-AKI

Cardiac surgery acute kidney injury (CS-AKI) postoperatively involves three major processes. Firstly, hypoperfusion which may impair perioperative renal oxygen delivery [1] reflected in increased heart-type fatty acid-binding protein (H-FABP) [2] and vascular endothelial growth factor (VEGF) [3]. Secondly, ischemia reperfusion injury (IRI) (reflected in increased midkine (MK) [4]) accentuates intrarenal inflammatory processes [5], and MK deficient mice have been shown to have resistance to renal IRI [6]. Thirdly, additional pro-inflammatory injury may arise from filtered reno-toxic pro-inflammatory biomarkers generated by the surgical trauma and the cardiopulmonary bypass (CPB) process [7]. This is reflected in increased plasma tumor necrosis factor soluble receptor 1 (pTNFsr1) and plasma tumor necrosis factor soluble receptor 2 (pTNFsr2) [8] which may be considered as surrogate biomarkers for the underlying blood pro-inflammatory response [9]. Accordingly, in preliminary analysis of serum samples of 401 patients undergoing elective cardiac surgery, logistic regression analyses confirmed that biomarkers predictive of postoperative renal dysfunction represented the three underlying processes: hypoperfusion (H-FABP), IRI (MK) and pro-inflammation (serum tumor necrosis factor soluble receptor 1 (sTNFsr1) and serum tumor necrosis factor soluble receptor 2 (sTNFsr2)) [9].

### Mechanisms protecting against CS-AKI

In contrast to the three major injurious processes identified in blood samples, a possible protective role against CS-AKI has been attributed to an intra renal anti-inflammatory cytokine response at cardiac surgery, characterized by perioperative increases in urinary tumor necrosis factor soluble receptor 2 (uTNFsr2) and urinary interleukin-1 receptor antagonist (uIL-1RA) [7, 10–12]. The urinary anti-inflammatory uTNFsr2 response precedes and greatly exceeds the corresponding plasma anti-inflammatory perioperative response [10]. Moreover, the molecular weights of uIL-1RA, urinary tumor necrosis factor soluble receptor 1 (uTNFsr1) and uTNFsr2, are greater than 20 kDa and therefore, their glomerular filtration is lower than their smaller pro-inflammatory counterparts [13]. These considerations point to urinary anti-inflammatory biomarkers being mainly generated within the kidney with a smaller additional contribution from glomerular filtration [10, 11]. Therefore, plasma levels of pTNFsr1, pTNFsr2 and plasma interleukin-1 receptor antagonist (pIL-1RA) are constitutively higher than the normal detectable levels of tumor necrosis factor α (TNFα) and interleukin-1α (IL-1α) which are readily filtered. Previously McBride et al. [7] demonstrated that post-operative CS-AKI patients had lower uIL-1RA and uTNFsr2 responses than non-CS-AKI patients prompting suggestions of an inadequate urinary anti-inflammatory response heightening vulnerability to CS-AKI. However, the study did not evaluate how urinary anti-inflammatory/pro-inflammatory balance affected CS-AKI development as key filtered pro-inflammatory biomarkers (interleukin-1β (IL-1β) and TNFα) involved in tubular injury in vitro [14] were not meaningfully detectable in normal urine [15] since they are thought to be absorbed by the tubules [16]. Urinary pro-inflammatory biomarkers are now measurable e.g., interferon gamma-induced protein-10 (IP-10), monocyte chemotactic protein-1 (MCP-1) and macrophage inflammatory protein-1δ (MIP-1δ) in diabetic nephropathy patients [15]; neutrophil gelatinase-associated lipocalin (NGAL), macrophage inflammatory protein–1α (MIP-1α) and IP-10 in CS-AKI patients [1, 3, 17]; MCP-1 in mice with AKI [18] and interleukin-12 subunit p40 (IL-12p40) in type 1 diabetes [19]. The renal impact of urinary anti- and pro-inflammatory balance is currently unknown.

Although hypoperfusion and IRI are directly injurious to the kidneys [6], their ability to indirectly injure kidneys through augmenting the pro-inflammatory response [20, 21] raises the question whether the urinary anti-inflammatory response also protects against hypoperfusion and IRI. Therefore, we investigated how urinary anti-inflammatory biomarkers/hypoperfusion biomarkers and urinary anti-inflammatory biomarkers/IRI biomarkers affected CS-AKI development.

### Study aims

De novo measurements of blood pro-inflammatory biomarkers and urinary pro- and anti-inflammatory biomarkers were analyzed together with the previously reported data on serum biomarkers of inflammation, hypoperfusion and IRI [9] to determine the following hypothesis.

### Detailed hypothesis

We investigated the primary hypothesis that CS-AKI development involves:

- (Ia) Higher blood pro-inflammatory response in parallel with
- (Ib) Lower blood anti-inflammatory response
- (Ic) The ratio of blood anti-inflammatory/pro-inflammatory biomarkers would be lower in CS-AKI than non-CS-AKI

The secondary hypothesis postulates that:

- (IIa) CS-AKI patients exhibit higher urinary pro-inflammatory response in parallel with
- (IIb) Lower urinary anti-inflammatory response
- (IIc) The ratio of urinary anti-inflammatory/pro-inflammatory biomarkers and the ratio of
- (IId) Urinary anti-inflammatory/blood pro-inflammatory biomarkers would be lower in CS-AKI than non-CS-AKI patients

Since hypoperfusion is an important contributing factor to CS-AKI, H-FABP and VEGF was measured in all cases. It was anticipated that the insult of hypoperfusion would be mitigated in patients who managed to mount a satisfactory urinary anti-inflammatory response. Accordingly, our third hypothesis was:

- (IIIa) Pre and post-operative H-FABP and VEGF would be higher in CS-AKI patients and
- (IIIb) The ratio of urinary anti-inflammatory biomarkers/serum hypoperfusion biomarkers (H-FABP and VEGF) would be lower in the CS-AKI patients
- Finally, since IRI is an important mechanism in CS-AKI, we hypothesized that
- (IVa) Midkine in blood and
- (IVb) Urine, would be higher in CS-AKI patients and the ratios of urinary anti-inflammatory biomarkers/serum midkine would be lower in CS-AKI patients

## Methods

Details of patient recruitment, demographics, inclusion and exclusion criteria, anesthetic technique, sample collection and processing, laboratory analysis of biomarkers have been described previously [9]. In addition to serum and plasma, urine samples were also collected from all patients. Urine samples were aliquoted and frozen at − 80 °C. Patient samples were analyzed using Randox cytokine arrays and run on an Evidence Investigator, as per manufacturer’s instructions (Randox Laboratories Ltd., Crumlin, Northern Ireland).

Briefly, 401 consecutive patients (*n* = 401) undergoing elective cardiac surgery were included in the study. Patients were recruited within the Cardiac Surgical Unit of the Royal Victoria Hospital Belfast, Northern Ireland. Local ethical committee and institutional approvals were received and written informed patient consent was obtained. Exclusion criteria included preoperative dialysis-dependent renal failure or significant renal disease (estimated glomerular filtration rate eGFR< 40) and diabetes mellitus. Patients on preoperative angiotensin conversion enzyme (ACE) inhibitor therapy were not excluded. The anesthetic technique was based on the use of propofol and fentanyl. Isoflurane was used in most patients either as an adjunct anesthetic agent or to control blood pressure. Pancuronium provided muscle relaxation. Postoperative analgesia was with morphine infusion.

### Definitions of renal dysfunction used in the study

Using the risk, injury, failure, loss, end-stage (RIFLE) Criteria of Renal Dysfunction [22, 23] CS-AKI was defined as in our earlier study [7] as a drop from baseline eGFR of greater than 25% (as calculated by the method of modification of diet in renal disease (MDRD) [24, 25] occurring within the first 24 and 48 postoperative hours (early renal dysfunction), on the fifth postoperative day (late renal dysfunction) or at any time throughout the 5-day postoperative period (early and late combined).

### Cytokine analysis

Cytokines that were studied in blood and urine are described in the Additional file 1.

### Statistical analysis

Statistical analysis was performed using SPSS ver. 25 (IBM). Mann Whitney U test was used to identify significant biomarkers. Biomarkers with a *p* < 0.05 were considered significant. Area Under Receiver Operating Characteristic (AUROC) was used as a measure for predictive ability for cytokine(s) or cytokine ratio(s) comparing non-CS-AKI and CS-AKI populations in the study. Cytokine ratios were calculated and ratios with the highest predictive ability (AUROC) to identify patients at risk of developing CS-AKI Any Day, pre, and post cardiac surgery, were investigated. Backward and Forward Wald and Forced Entry logistic regression was used to identify the ratio combinations with the highest predictive ability (AUROC) to identify CS-AKI patients.

## Results

### Patient demographics

Full details of demographic data, distribution of surgical procedures, postoperative management and major outcomes have been previously described [9]. Patient characteristics are described in Table 1.

**Table 1:** Clinical characteristics of the study patients

	non-CS-AKI (***n*** = 273)	CS-AKI (***n*** = 71)	***P*** value
Age (years)	65.4 ± 11.6	68.6 ± 10.7	0.020
Gender (male)	192/273 (70.3%)	50/71 (70.4%)	0.988
Weight (kg)	80.9 ± 17.5	84.8 ± 16.6	0.061
Height (cm)	167.8 ± 11.4	165.1 ± 14.0	0.082
BMI (kg/m^2^)	28.9 ± 10.2	31.0 ± 6.0	0.001
Diabetes	29/268 (10.8%)	16/68 (23.5%)	0.006

*CS-AKI* Cardiac surgery acute kidney injury, *n* Number of patients, *BMI* Body mass index

### Results for CS-AKI in comparison with non-CS-AKI patients

#### Hypothesis I-a (blood pro-inflammatory biomarkers)

Compared with non-CS-AKI patients, blood pro-inflammatory biomarkers were increased both pre- and postoperatively in patients with CS-AKI identified at Any Day (Table 2) and Days 1, 2, 5 (Supplemental Table 1, 3, 5, respectively).

**Table 2:** Blood pro-inflammatory and anti-inflammatory cytokines

Blood cytokines	Pre-op or post-op	Any day						
		non-CS-AKI			CS-AKI			***P*** value
		n	median	IQR	n	median	IQR	
**Pro-inflammatory cytokines**								
sIP-10	pre-op	260	113.10	(84.93–165.81)	64	133.83	(100.77–208.24)	0.012
sIP-10	post-op	254	101.82	(62.42–174.68)	65	125.87	(80.45–206.44)	0.046
sIL-12p40	pre-op	260	368.87	(268.61–582.54)	64	569.18	(390.29–874.85)	< 0.001
sIL-12p40	post-op	254	262.82	(179.13–397.17)	65	442.36	(297.27–615.77)	< 0.001
sMK	post-op	212	979.28	(474.26–2185.78)	58	2365.00	(1074.5–4306.67)	< 0.001
pIL-6	pre-op	254	2.17	(1.39–4.03)	64	3.03	(1.93–7.69)	0.001
pIL-8	post-op	259	8.54	(5.74–13.65)	65	11.82	(6.84–19.38)	0.005
pMIP-1α	pre-op	251	3.53	(2.79–4.37)	64	4.52	(3.03–5.55)	0.001
pMIP-1α	post-op	256	4.33	(3.33–6.38)	65	6.63	(4.33–10.12)	< 0.001
pMCP-1	pre-op	254	129.00	(105–152)	64	143.00	(110.75–171.75)	0.008
pMCP-1	post-op	259	194.00	(139–274)	65	248.00	(183.5–350)	0.001
pNGAL	pre-op	255	572.89	(437.04–742.77)	64	672.05	(564.33–951.71)	0.001
pNGAL	post op	260	948.07	(646.34–1300.38)	65	1439.01	(1036.43–1902.86)	< 0.001
pTNFα	pre-op	254	2.05	(1.66–2.54)	64	2.50	(2–2.89)	< 0.001
pTNFα	post-op	259	2.33	(1.8–3.17)	65	3.11	(2.06–4.45)	< 0.001
**Anti-inflammatory cytokines**								
sTNFsr1	pre-op	260	0.33	(0.26–0.45)	64	0.52	(0.37–0.68)	< 0.001
sTNFsr1	post-op	254	0.68	(0.52–0.85)	65	1.04	(0.79–1.4)	< 0.001
sTNFsr2	pre-op	260	0.35	(0.24–0.56)	64	0.57	(0.37–0.85)	< 0.001
sTNFsr2	post-op	254	0.71	(0.49–0.99)	65	1.25	(0.77–1.66)	< 0.001
sIL-1RA	pre-op	260	60.80	(40.63–105.57)	64	81.99	(55.14–132.15)	0.005
sIL-1RA	post-op	254	406.48	(242.34–794.63)	65	739.25	(440.03–1887)	< 0.001
pIL-10	post-op	259	2.27	(1.48–4.64)	65	3.27	(1.85–5.69)	0.009

*pre-op* Preoperative, *post-op* Postoperative, *CS-AKI* Cardiac surgery acute kidney injury, *n* Number of patients, *sIP-10* Serum interferon gamma - induced protein-10, *sIL-12p40* Serum interleukin-12 subunit p40, *sMK* Serum midkine, *pIL-6* Plasma interleukin-6, *pIL-8* Plasma interleukin-8, *pMIP-1α* Plasma macrophage inflammatory protein-1α, *pMCP-1* Plasma monocyte chemotactic protein-1, *pNGAL* Plasma neutrophil gelatinase-associated lipocalin, *pTNFα* Plasma tumor necrosis factor α, *sTNFsr1* Serum tumor necrosis factor soluble receptor 1, *sTNFsr2* Serum tumor necrosis factor soluble receptor 1, *sIL-1RA* Serum interleukin-1 receptor antagonist, *pIL-10* Plasma interleukin-10

#### Hypothesis I-b (blood anti-inflammatory biomarkers)

Pre- and postoperative sTNFsr1, sTNFsr2 and sIL-1ra were lower in non-CS-AKI patients than CS-AKI identified at Any Day (Table 2), Days 1 and 2 (Supplemental Table 1 and 3). Some cytokines were increased at Day 5 (Supplemental Table 5).

#### Hypothesis I-c (ratios of blood anti-inflammatory/blood pro-inflammatory biomarkers)

Ratios were higher in both pre- and post-operative CS-AKI patients compared with non-CS-AKI patients identified at Days 1, 2, 5 and Any Day (Supplemental Table 7, 13, 19, 25, respectively).

#### Hypothesis II-a (urinary pro-inflammatory biomarkers)

Pre- and postoperative uIL-12p40; postoperative uIP-10 and uNGAL were higher in Any Day CS-AKI patients (Table 3). Several cytokines were raised in Days 1, 2 and 5 CS-AKI patients (Supplemental Table 2, 4, 6, respectively).

**Table 3:** Urinary pro-inflammatory and anti-inflammatory cytokines

Urinary cytokines	Pre-op or post-op	Any day						
		non-CS-AKI			CS-AKI			***P*** value
		n	median	IQR	n	median	IQR	
**Pro-inflammatory cytokines**								
uIP-10	post-op	256	12.68	(6.31–25.29)	65	22.97	(9.71–61.26)	< 0.001
uIL-12p40	pre-op	258	0.94	(0–3.34)	65	2.53	(0–4.29)	0.035
uIL-12p40	post-op	256	2.87	(0–4.31)	65	4.47	(2.95–7.23)	< 0.001
uNGAL	post-op	247	135.09	(68.7–306.8)	64	234.44	(136.94–429.46)	< 0.001
**Anti-inflammatory cytokines**								
uTNFsr1	pre-op	257	0.53	(0.3–0.87)	65	0.70	(0.42–1.07)	0.044
uTNFsr1	post-op	254	6.66	(4.4–8.71)	65	7.70	(5.31–10.28)	0.020
uTNFsr2	pre-op	257	0.83	(0.34–1.52)	65	1.26	(0.48–2.06)	0.020
uTNFsr2	post-op	255	8.08	(5.9–10.08)	65	9.56	(6.41–12.3)	0.018
uIL-1RA	post-op	247	8616.30	(3828.3–18,027.3)	64	13,274.55	(5696.63–31,703.93)	0.024

*pre-op* Preoperative, *post-op* Postoperative, *CS-AKI* Cardiac surgery acute kidney injury, *n* Number of patients, *uIP-10* Urinary interferon gamma - induced protein-10, *uIL-12p40* Urinary interleukin-12 subunit p40, *uNGAL* Urinary neutrophil gelatinase-associated lipocalin, *uTNFsr1* Urinary tumor necrosis factor soluble receptor 1, *uTNFsr2* Urinary tumor necrosis factor soluble receptor 2, *uIL-1RA* Urinary interleukin-1 receptor antagonist

#### Hypothesis II-b (urinary anti-inflammatory biomarkers)

Pre- and postoperative uTNFsr1, uTNFsr2 and postoperative uIL-1RA were significantly elevated in Any Day CS-AKI patients (Table 3). On Day 1, only postoperative uTNFsr2 was significantly increased in CS-AKI patients (Supplemental Table 2) whereas on Day 2 preoperative uTNFsr1 was increased (Supplemental Table 4). At Day 5 there was no significant differences in the level of urinary anti-inflammatory cytokines between non-CS-AKI and CS-AKI patients.

#### Hypothesis II-c (ratios of urinary anti-inflammatory/urinary pro-inflammatory biomarkers)

Several postoperative ratios were significantly lower in the Any Day, Days 1, 2 and 5 CS-AKI patients (Supplemental Table 8, 14, 20 and 26, respectively).

#### Hypothesis II-d (ratios of urinary anti-inflammatory/blood pro-inflammatory biomarkers)

Pre- and postoperative uIL-1RA/pIL-6, uIL-1RA/sIL-12p40, uIL-1RA/pMIP-1α, uIL-1RA/pNGAL and eleven other postoperative ratios were significantly lower in Any Day CS-AKI patients (Supplemental Table 27). On Day 1, preoperatively only one ratio uIL-1RA/ pIL-6 was significantly lower in CS-AKI patients whereas postoperatively eight ratios were significantly lower (Supplemental Table 9).

#### Hypothesis III-a (Hypoperfusion biomarkers - VEGF and H-FABP)

Preoperative VEGF and postoperative H-FABP were significantly elevated on Days 1, 2, 5 and Any Day CS-AKI patients (Supplemental Tables 10, 16, 22, 28, respectively). Preoperative H-FABP was significantly increased on Day 1, 2 and Any Day in CS-AKI patients (Supplemental Tables 10, 16 and 28, respectively).

#### Hypothesis III-b (ratios of urinary anti-inflammatory/blood hypoperfusion biomarkers)

Preoperative uIL-1RA/pVEGF was significantly lower only in Day 5 in CS-AKI patients (Supplemental Table 23). Postoperative uTNFsr1/sH-FABP and uTNFsr2/sH-FABP were significantly lower in Day 1, 2, 5 and Any Day in CS-AKI patients (Supplemental Tables 11, 17, 23, 29, respectively). Pre- and postoperative uIL-1RA/sH-FABP was significantly lower in Day 2 and Any Day CS-AKI patients (Supplemental Tables 17 and 29, respectively).

#### Hypothesis IV (serum MK elevated post-operatively in CS-AKI patients)

Serum (but not urinary) MK post-operatively was elevated in Days 1, 2 and 5 in CS-AKI patients. Moreover, postoperatively ratios of uIL-1RA, uTNFsr1 and uTNFsr2 with sMK were lower in Days 1, 2 and 5 in CS-AKI patients.

#### Logistic regression models

**Fig. 1:**
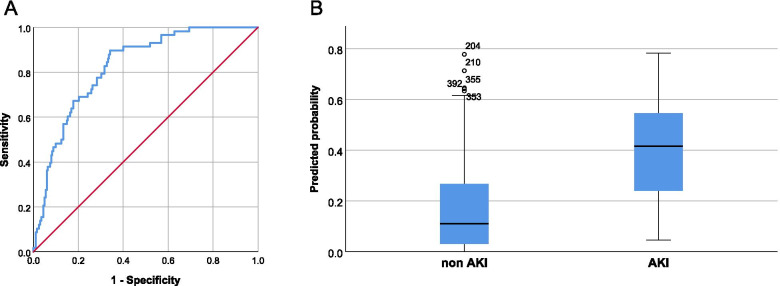
Logistic Regression Models. The model with the highest predictive ability to identify patients at risk of developing CS-AKI Any Day postoperatively was uIL-1RA/sIL-12p40 + uTNFsr2/sH-FABP + uIL-1RA/sMK. **A** Postoperative model of biomarkers used to distinguish between non-CS-AKI and CS-AKI patients (AUROC 0.824). **B** Predicted probability of the model to identify non-CS-AKI and CS-AKI patients

Preoperatively the ratio of uIL-1RA/pIL-6 had the highest predictive ability (AUROC 0.616) to identify postoperative CS-AKI (Table 4). However, postoperatively the combination of ratios uIL-1RA/sIL-12p40 + uTNFsr2/sH-FABP + uIL-1RA/sMK (Any Day) had the highest predictive ability to identify patients at risk of developing CS-AKI with AUROC 0.824 (Table 4, Fig. 1A and B).

**Table 4:** Cytokine ratios with the highest predictive ability to identify patients at risk of developing CS-AKI

Any Day					
	Biomarker(s)	AUROC	CI	Sensitivity	Specificity
Preoperative	uIL-1RA/pIL-6	0.616	0.538–0.694	67.2%	52.4%
Postoperative	uIL-1RA/sIL-12p40 + uTNFsr2/sH-FABP + uIL-1RA/sMK	0.824	0.770–0.879	82.8%	67.8%
	uIL-1RA/sIL-12p40 + uTNFsr2/sH-FABP	0.775	0.715–0.835	72.3%	67.1%
	uIL-1RA/sIL-12p40 + uIL-1RA/sMK	0.784	0.720–0.848	74.1%	69.1%
	uTNFsr2/sH-FABP + uIL-1RA/sMK	0.771	0.709–0.832	74.1%	68.6%
	uIL-1RA/sIL-12p40	0.725	0.658–0.792	72.3%	63.9%
	uTNFsr2/sH-FABP	0.685	0.617–0.753	73.8%	56.6%
	uIL-1RA/sMK	0.704	0.632–0.776	74.1%	55.3%

*CS-AKI* Cardiac surgery acute kidney injury, *AUROC* Area under receiver operating characteristic, *CI* Confidence interval, *uIL-1RA* Urinary interleukin-1 receptor antagonist, *pIL-6* Plasma interleukin-6, *sIL-12p40* Serum interleukin-12 subunit p40, *uTNFsr2* Urinary tumor necrosis factor soluble receptor 2, *sH-FABP* Serum heart-type fatty acid-binding protein, *sMK* Serum midkine

## Discussion

Our main findings are that a heightened urinary anti-inflammatory response appears to be protective against the renal injurious effects of a pro-inflammatory response, IRI and hypotension. With respect to each of the hypotheses beginning with result (I-a), this is consistent with existing evidence of the perioperative pro-inflammatory response being injurious to kidneys. Furthermore, presence of elevated concentrations of preoperative blood pro-inflammatory mediators in those who later developed CS-AKI suggests that preoperative drivers of pro-inflammation are important in subsequent pathogenesis of CS-AKI.

Findings to hypotheses I-b and I-c suggest that the greater blood pro-inflammatory response in the CS-AKI patients (I-a) is accompanied by a proportionately greater blood anti-inflammatory response (I-b and I-c) and despite this compensatory response, CS-AKI ensued. This would suggest that the blood anti-inflammatory response is insufficient to neutralize the harmful effects of the blood pro-inflammatory response as far as blood related pro-inflammatory-mediated renal injury is concerned. This may be because the blood pro-inflammatory biomarkers are of lower molecular weight than their anti-inflammatory counterparts, such that the former are more easily filtered by the glomerulus and thus, enter the glomerular filtrate without the same protective anti-inflammatory constraints they face in the blood [13]**.** Furthermore, a separate intrarenal anti-inflammatory mechanism, may have failed in CS-AKI patients. Arguably, this is likely if there was support for the secondary hypothesis anticipating a higher urinary pro-inflammatory response (II-a) accompanied by an inadequate urinary anti-inflammatory response (II-b, II-c) in CS-AKI than non-CS-AKI patients. In comparison with patients who never developed renal dysfunction postoperatively, consistent with the secondary hypothesis (II-a), patients who developed CS-AKI, had higher pre- and postoperative urinary concentrations of pro-inflammatory biomarkers (e.g., pre- and postoperative uIL-12p40; postoperative uNGAL, uIP-10).

In contrast with the secondary hypothesis (II-b), which was based on the demonstration of a significantly reduced urinary anti-inflammatory response in CS-AKI patients in the 2013 study [7], patients who developed CS-AKI in the present study had higher pre- and postoperative urinary concentrations of anti-inflammatory biomarkers (pre- and postoperative uTNFsr1, uTNFsr2; postoperative uIL-1RA).

Since the publication of the 2013 study [7], clinical practice has changed in the ICU at the Royal Victoria Hospital Belfast such that routine use of noradrenaline has increased to provide consistently higher perioperative blood pressures particularly during CPB. In the 2013 study [7] only 10.6% of all patients were on noradrenaline perioperatively whereas in this study 60% of patients received noradrenaline. In neither study was there a difference in noradrenaline use between CS-AKI and non-CS-AKI groups. It could be argued that the increased noradrenaline use in the present study, could increase perioperative blood pressure and reduce perioperative hypotensive episodes sufficiently to lead to somewhat enhanced filtration of the relatively difficult to filter, large molecular weight blood anti-inflammatory biomarkers (TNFsr1, TNFsr2, IL-1RA). However, it was possible that in the 2013 study [7], due to less noradrenaline usage, mean arterial pressures may have been lower perioperatively. This would cause reduced filtration of the perioperatively increased blood anti-inflammatory biomarkers, resulting in changes in the magnitude of urinary anti-inflammatory biomarkers, since the latter arose largely from endogenous intrarenal generation of urinary anti-inflammatory biomarkers without a significant top up from filtered plasma biomarkers. Therefore, lower levels of urinary anti-inflammatory biomarkers in CS-AKI patients noted in the 2013 study [7] reflected an inadequate intrarenal generation of anti-inflammatory biomarkers. From the greater use of noradrenaline in the present study, it is possible that measurements of urinary anti-inflammatory biomarkers reflect not only intrarenally-generated biomarkers, but also a heightened contribution from filtered blood anti-inflammatory biomarkers serving to top up the intrarenally-generated anti-inflammatory cytokines.

In the present study, blood anti-inflammatory biomarkers are of higher concentration in CS-AKI than non-CS-AKI patients. Any improvement in filtration of these blood cytokines due to greater noradrenaline usage, could lead to an augmentation of the intrarenally-generated anti-inflammatory response. Since blood anti-inflammatory biomarkers are raised in CS-AKI than non-CS-AKI, this could explain the increased urinary concentrations of these biomarkers in the CS-AKI group compared with non-CS-AKI (finding II-b). Furthermore, any improvement is likely to be accompanied by an even greater increase in filtration of blood pro-inflammatory biomarkers, although this is difficult to measure directly. This may explain increased urinary anti-inflammatory biomarkers in CS-AKI than non-CS-AKI patients in this study, and yet despite this, such increases seemed inadequate to confer renal protective advantage. The proportion of filtered anti-inflammatory biomarkers was not commensurate with the filtered pro-inflammatory biomarkers as indirectly measured by urinary pro-inflammatory biomarkers. However, in patients who can develop an adequate intrarenally-generated anti-inflammatory response, protection against pro-inflammatory biomarkers is conferred.

This leads to the key finding in the present study, namely confirmation of the secondary hypotheses II-c and II-d, that patients who developed CS-AKI had lower ratios of urinary anti−/pro-inflammatory biomarkers (II-c) and urinary anti−/blood pro-inflammatory biomarkers (II-d).

Findings II-a and II-b suggest that although the urinary pro- (II-a) and anti-inflammatory (II-b) responses were greater in CS-AKI than non-CS-AKI patients, in contrast to blood, urinary anti−/pro-inflammatory ratios (II-c) and urinary anti−/blood pro- inflammatory ratios (II-d) were consistently lower (II-c and II-d) in CS-AKI than non-CS-AKI patients. This suggests that in CS-AKI patients, while there is a measure of compensatory anti-inflammatory activity in urine (II-b), in contrast to blood (I-b), this intrarenal anti-inflammatory response is of inadequate magnitude (II-c and II-d) to protect against inflammatory-mediated CS-AKI. This work suggests, for the first time, that a compromised intrarenal anti-inflammatory response constitutes an important and until now, undescribed mechanism involved in the pathogenesis of CS-AKI.

We hypothesized that an anti-inflammatory response in blood and urine adequately protected against a hypoperfusion insult indicated by increased plasma VEGF and serum H-FABP. Plasma VEGF preoperatively and serum H-FABP pre- and postoperatively were higher in CS-AKI than non-CS-AKI patients (III-a). However, this risk can be reduced where there are compensatory increases in pre- and postoperative uIL-1RA and postoperative uTNFsr1 and uTNFsr2.

MK is a marker of IRI associated with CS-AKI [9]. Since pro-inflammatory cytokine generation is an important aspect of IRI [20] we evaluated if the ratio of urinary anti-inflammatory cytokine/serum MK was lower in CS-AKI patients. In any estimation of pro- and anti-inflammatory cytokine balance in blood and urine, the magnitude of change in concentrations of blood and urinary anti-inflammatory biomarkers is greater than changes in blood and urinary pro-inflammatory biomarkers which are generally at lower concentrations. For example, concentrations of blood TNFα and IL-1β and changes in their level are small in comparison with serum TNFsr1, TNFsr2 and IL-1RA. Moreover, filtered TNFα and IL-1β are normally undetectable in the urine (as they are absorbed and destroyed by the tubules [16]). Therefore, surrogates for urinary TNFα were used, namely uIP-10 and uIL-12p40 whose presence in urine may reflect TNFα activity more proximally in the filtrate. However, if it is assumed that the sTNFsr1 and sTNFsr2 is an adequate compensatory response to transient, small increases in blood TNFα, and serum IL-1RA is an adequate response to blood IL-1β, then sTNFsr1 and sTNFsr2 could be taken as surrogates for filtered TNFα, whereas IL-1RA is a surrogate for filtered IL-1β. Therefore, the ratios of sTNFsr1, sTNFsr2, sIL-1RA, uTNFsr1, uTNFsr2 and uIL-1RA were investigated. This is effectively a ratio of filtered blood pro-inflammatory biomarker/urinary anti-inflammatory biomarker, if serum TNFsr1 and TNFsr2 are surrogates for filtered serum TNFα and urinary IL-1RA a surrogate for filtered IL-1β. Postoperative ratios sTNFsr1/uTNFsr2, sTNFsr2/uTNFsr2, sIL-1RA/uTNFsr2, sTNFsr1/uTNFsr1, sTNFsr2/uTNFsr1, sIL-1RA/uTNFsr1 were all significantly higher in Day 5 CS-AKI patients. The data suggests that the urinary anti-inflammatory response is protective against filtered pro-inflammatory biomarkers.

### Clinical application

**Fig. 2:**
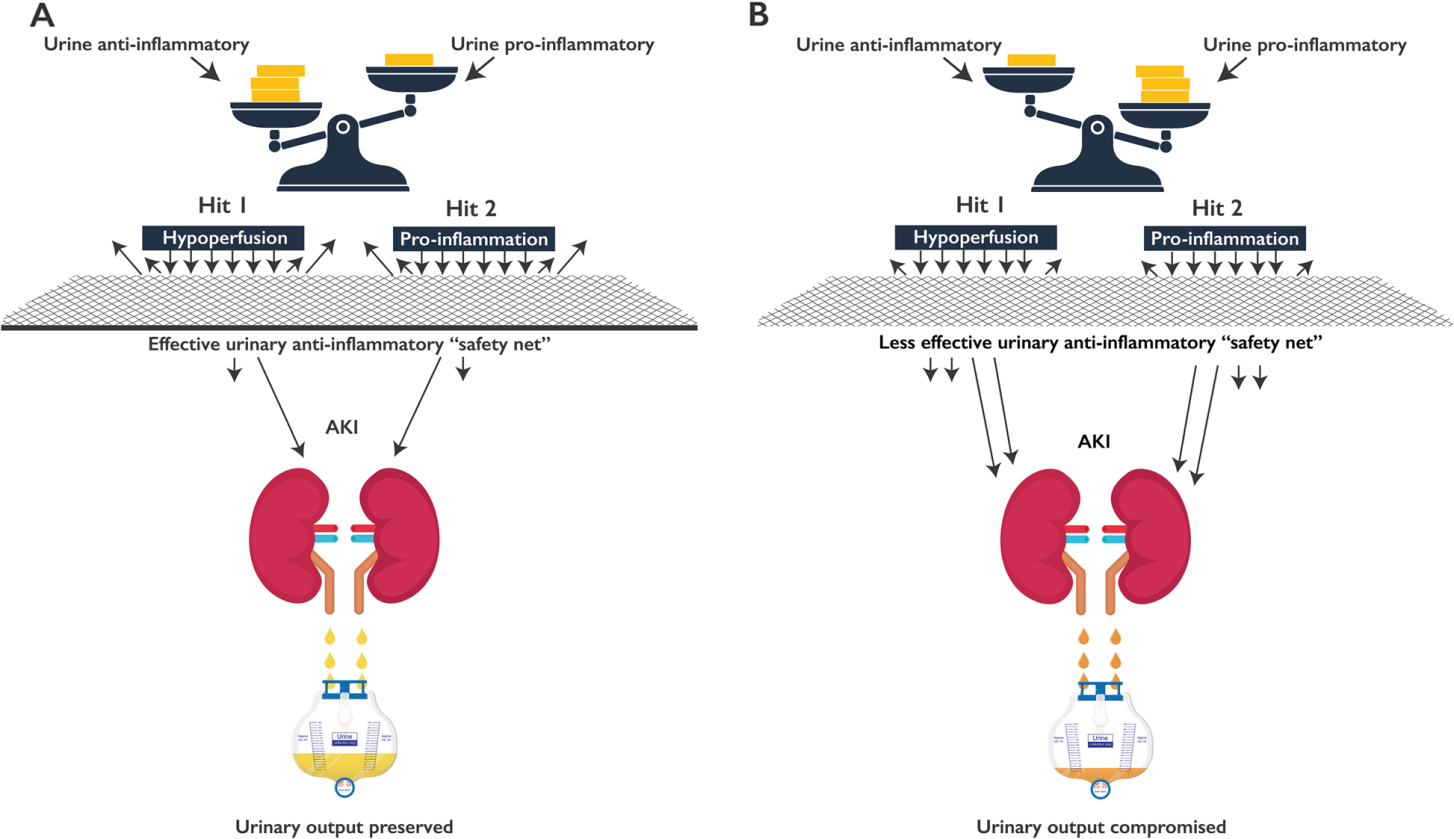
Potential pathways involved in the pathogenesis of AKI. Heightened urinary anti-inflammatory response protects against renal injury caused by pro-inflammation and hypoperfusion. **A** When urinary anti-inflammatory response is proportionally greater than urinary pro-inflammatory response, the balance of urinary anti−/pro-inflammatory biomarkers is in favor of anti-inflammation. **B** However, when the response is smaller, there is less protection against pro-inflammation and hypoperfusion, the balance is less favorable with respect to anti-inflammation

When the above blood and urinary ratios were subjected to Backward Wald and Forced Entry logistic regression, preoperative Any Day ratio uIL-1RA/pIL-6 had the highest predictive ability to identify postoperative CS-AKI with AUROC 0.616. However, postoperatively the combination of Any Day ratios uIL-1RA/sIL-12p40 + uTNFsr2/sH-FABP + uIL-1RA/sMK had the highest predictive ability to identify patients at risk of developing CS-AKI with AUROC 0.824 (Table 4, Fig. 1a and b). The results illustrate the imbalance between postoperative urinary anti-inflammation and three factors: (i) blood pro-inflammation (uIL-1RA/sIL-12p40), (ii) hypoperfusion (uTNFsr2/sH-FABP) and (iii) IRI (uIL-1RA/sMK). This model reflects the underlying concept of urinary anti-inflammatory response protecting against the three insults of perioperative pro-inflammation, hypoperfusion and IRI (Fig. 2). This information can be used to inform clinical management in the areas of treatment choices and planning intraoperative management.

Figure 2 is an original drawing created by the authors of the manuscript who permit reproduction by BMC Nephrology.

### Treatment choices and planning

Increasing use of minimally invasive techniques (e.g. mini-sternotomy, robotic surgery), allow a reduced perioperative pro-inflammatory response as compared with traditional cardiac surgery. These options may be more attractive in patients where preoperatively an inadequate urinary anti-inflammatory response has been identified. Furthermore, since an inadequate urinary anti-inflammatory response renders the patient more vulnerable to hypoperfusion, a treatment option involving temporary permissive hypoperfusion such as off pump coronary artery bypass surgery would not be advisable in someone with an inadequate urinary anti-inflammatory response.

### Intraoperative management

More studies are needed to confirm if managing patients perioperatively on higher than normal perfusion pressures improves the urinary anti-inflammatory response. However, it could be argued that preoperative identification of patients exhibiting inadequate urinary anti-inflammatory levels and a resulting reduction in protection in subsequent hypoperfusion, could result in these vulnerable patients being managed at supra-normal perioperative blood pressures to avoid hypoperfusion (even though this may make the surgical field more challenging for the operator).

### Post-operative care

A post-operatively identified unfavorable urinary anti-inflammatory response may be taken as a contraindication to non-steroidal anti-inflammatory analgesics.

In the long term, these findings may help identify at risk patient populations who could be enrolled in interventive trials. One concept in need of testing is the effectiveness of deploying strategies to precondition the anti-inflammatory response of patients found preoperatively to have deficient anti-inflammatory protection.

## Conclusions

In summary, this study has demonstrated that the three main causes of perioperative CS-AKI, namely inflammation, hypoperfusion and ischemia/reperfusion injury, are countered by a urinary anti-inflammatory response. Moreover, ratios of blood anti-inflammatory/pro-inflammatory biomarkers, urinary anti-inflammatory/pro-inflammatory biomarkers, urinary anti-inflammatory/blood anti-inflammatory biomarkers, urinary anti-inflammatory/hypoperfusion biomarkers and urinary anti-inflammatory/IRI biomarkers have predictive utility. Further work is required to determine how these parameters, effective in predicting renal dysfunction in elective cardiac surgery, will find application in other contexts such as trauma, general surgery and sepsis. This work has potential relevance in preoperatively selecting patients who could benefit from targeted therapeutic strategies which could be developed to enhance under-functioning endogenous anti-inflammatory intrarenal protective mechanisms to maximize a patient’s ability to counter perioperative pro-inflammatory, hypoperfusion and IRI insults.

## Supplementary Information

**Additional file 1. **Detailed justification of mediators chosen in this study.

**Additional file 2. **Supplementary data, Tables 1–30.

## Data Availability

The dataset used and/or analyzed during the current study are available from the corresponding author on reasonable request.

## Abbreviations

CS-AKI: Cardiac surgery acute kidney injury
H-FABP: Heart-type fatty acid-binding protein
VEGF: Vascular endothelial growth factor
IRI: Ischemia reperfusion injury
MK: Midkine
pTNFsr1: Plasma tumor necrosis factor soluble receptor 1
pTNFsr2: Plasma tumor necrosis factor soluble receptor 2
sTNFsr1: Serum tumor necrosis factor soluble receptor 1
sTNFsr2: Serum tumor necrosis factor soluble receptor 2
uTNFsr2: Urinary tumor necrosis factor soluble receptor 2
uIL-1RA: Urinary interleukin-1 receptor antagonist
uTNFsr1: Urinary tumor necrosis factor soluble receptor 1
pIL-1RA: Plasma interleukin-1 receptor antagonist
TNFα: Tumor necrosis factor α
IL-1α: Interleukin-1α
IL-1β: Interleukin-1β
IP-10 (CXCL10): Interferon gamma - induced protein-10 (C-X-C motif chemokine 10)
MCP-1 (CCL2): Monocyte chemotactic protein-1 (chemokine (C-C motif) ligand 2)
MIP-1δ: Macrophage inflammatory protein-1δ
NGAL: Neutrophil gelatinase-associated lipocalin
MIP-1α (CCL3): Macrophage inflammatory protein-1α (chemokine (C-C motif) ligand 3)
IL-12p40: Interleukin-12 subunit p40
eGFR: Estimated glomerular filtration rate
ACE: Angiotensin converting enzyme
RIFLE: Risk, injury, failure, loss, end-stage
MDRD: Modification of diet in renal disease
AUROC: Area under receiver operating characteristic
IL-6: Interleukin-6
IL-8 (CXCL8): Interleukin-8 (chemokine (C-X-C motif) ligand 8)
TLR: Toll-like receptors
ICU: Intensive care unit
UTI: Urinary tract infection
MIP-2: Macrophage inflammatory protein-2
IL-12p35: Interleukin-12 subunit p35
IL-12p70 (IL-12): Interleukin-12 subunit p70
IL-23: Interleukin-23
CPB: Cardiopulmonary bypass
CK-MB: Creatine kinase-muscle/brain
AHF: Acute heart failure
HIF-1α: Hypoxia-inducible factor-1α
Pre-op: Preoperative
Post-op: Postoperative
