# Supplementary material for: Blood and urinary cytokine balance and renal outcomes at cardiac surgery
Source: BMC Nephrol. 2021 Dec 8;22:406. doi: 10.1186/s12882-021-02621-6 (PMC8653550; doi:10.1186/s12882-021-02621-6)
Supplement: Supplementary file 1 — Additional file 1. Detailed justification of mediators chosen in this study. [file 12882_2021_2621_MOESM1_ESM.docx]

**Detailed justification of mediators chosen in this study**

**Pro-inflammatory cytokines studied in blood and urine - TNF**α**, IL-1**β**, IL-6**

TNFα and IL-1β are directly injurious to *in vitro* cultured tubular cells (1). The levels of TNFα and IL-1β in plasma are slightly raised at cardiac surgery (2). A link between TNFα and AKI was observed in a murine model of sepsis-induced AKI, where pre-sepsis administered anti-TNFα antibodies protected against AKI development (3). Postoperative plasma TNFα predicted renal dysfunction following pediatric cardiac surgery (4). Furthermore, patients who develop AKI also have a higher level of interleukin-6 (IL-6) compared with non-AKI patients (5).

**Chemokines in the CXC & CC families studied in blood and urine**

Chemokines are produced due to IRI in the kidney (6) in addition to pro-inflammatory cytokines (7) both processes that are active in AKI. Chemokines are generally pro-inflammatory through selectively recruiting monocytes, neutrophils and lymphocytes. However, chemokines have multiple actions on non-immune cells e.g. IP-10 along with CXCL4, 9 and 11 activate chemokine receptor CXCR3 on vascular smooth muscle and endothelial cells, a process involved in atherosclerosis, hypertension and heart failure (8).

**IL-8 & IP-10**

Plasma interleukin-8 (IL-8) is produced in response to TNFα (7) and is elevated in cardiac surgery and isolated cardiopulmonary bypass experiments to concentrations higher than simultaneous rises of TNFα (2). This may suggest that its presence may reflect underlying TNFα activity during cardiac surgery. IL-8 levels are higher in trauma patients who develop AKI than those who do not (5). Raised preoperative plasma IL-8 levels predicted renal dysfunction following pediatric cardiac surgery (4).

IP-10 may already be more active in cardiac surgery patients than controls as it is implicated in pathogenesis of cardiovascular diseases including heart failure (8). IP-10 is secreted by monocytes, fibroblasts, endothelial and mesangial cells in response to interferon gamma and TNFα (7). In human mesangial cell culture, IP-10 is secreted by activation of Toll-like receptors (TLR) (9), along with TNFα, which in turn further amplifies IP-10 production in the kidney (10). IP-10 is detectable in urine (11,12) whereas TNFα and IL-1β are not (13), suggesting that IP-10 in the urine could be a useful surrogate for intrarenally produced TNFα and IL-1β, and thus is a useful biomarker of intrarenal inflammatory activity to compare with intrarenal anti-inflammatory activity – uTNFR1 and uTNFR2. In a paediatric study, urinary IP-10 expression was significantly increased in unilateral obstruction of the ureter on the first postoperative day (14). Also, in intensive care unit (ICU) patients, urinary concentrations of IP-10 were significantly higher in patients with AKI than those without AKI (15). Furthermore, since baseline blood levels of IP-10 are higher than that of TNFα and perioperative response of IP-10 is greater than that of TNFα, its measurement in blood may help to amplify small pro-inflammatory TNFα cytokine changes in blood to facilitate a more accurate assessment of blood or urinary pro- and anti-inflammatory balance.

**MCP-1 & MIP-1α**

Pro-inflammatory cytokines signal increased secretion of chemokines including MCP-1. Thus, MCP-1 is a downstream marker for an upstream pro-inflammatory cytokine response. Changes in blood pro-inflammatory cytokines are generally of small magnitude, therefore, the larger perioperative changes of MCP-1 in blood may help amplify small pro-inflammatory cytokine changes in blood to facilitate a more accurate assessment of blood pro- and anti-inflammatory balance. Peng et al (2015) demonstrated that expression of MCP-1 in a rat model of renal IRI was increased in the disease group even before alterations in serum creatinine and urea and, of all of the biomarkers measured, serum MCP-1 was the first to elevate, but rapidly returned to a normal baseline level (16). Several studies have confirmed MCP-1 modulation in murine IRI models (17–19). Bihorac et al (2013) (5) measured MCP-1 levels in patients following trauma and showed similar results to the murine models, finding that MCP-1 peaked in plasma in the early stages of AKI (5). Pro-inflammatory cytokines are almost undetectable in urine (13) therefore, measurment of urinary MCP-1 could be used as an indicator of upstream pro-inflammatory cytokine activity and thus, may help to assess the pro-inflammatory side of the pro-/anti-inflammatory balance in urine. In support of this argument, MCP-1 mRNA was observed to be upregulated and increased in urine in a rat model of renal ischemia/reperfusion injury (20,21).

Serum MIP-1α is elevated in trauma patients who go on to develop AKI (5,22) and in AKI secondary to malaria (23). MIP-1α is elevated in serum in urinary tract infection (UTI) vs. control (11). Since baseline blood levels of MIP-1α are higher than that of TNFα (11), and the perioperative response of MIP-1α is greater than that of TNFα, its measurement in blood may help to amplify small pro-inflammatory cytokine changes in blood to facilitate a more accurate assessment of blood pro- and anti-inflammatory balance. However, MIP-1α is also detectable in urine of AKI patients following cardiac surgery (12) but there was no differences in urinary concentrations between UTI and control (11).

**Blood and urinary Midkine**

Constitutive midkine (MK) gene expression in murine proximal tubules is increased with renal IRI (24) leading to induction of chemokines, such as macrophage inflammatory protein-2 (MIP-2) and MCP-1, a response less pronounced in MK(-/-) mice (24). The resulting increased infiltration of inflammatory cells cause severe tubulointerstitial injury (6). Serum MK is a sensitive and early marker of renal injury after contrast administration in low-risk patients undergoing percutaneous coronary interventions (25). Recently urinary MK has been linked with AKI at cardiac surgery (26).

**Blood and urinary IL-12p40**

IL-12p40 is pro-inflammatory cytokine (27) that forms a dimer with interleukin-12 subunit p35 (IL-12p35) to make interleukin-12 subunit p70 (IL-12p70). IL-12p40 is also a constituent of interleukin-23 (IL-23). In a murine model of increases in TNFα and IL-12p40, induced by scorpion venom, it was demonstrated that TNFα triggers the release of IL-12p40 (28). As TNFα is increased in patients with post-surgical AKI, and TNFα leads to production of IL-12p40, presence of IL-12p40 in blood and urine may reflect TNFα activity in blood and urine. Therefore, since TNFα levels are low in urine, and IL-12p40 is elevated (in ketamine abusers) (29), it follows that urinary IL-12p40 may be utilized as a surrogate for the urinary TNFα activity, in order to evaluate the balance of pro- and anti-pro-inflammatory activity in urine.

**NGAL**

In a meta-analysis of CS-AKI, plasma and urinary NGAL measured postoperatively accurately detects CS-AKI, especially in children (30). In contrast, uNGAL (peak day 1 or 2 post surgery) failed to accurately predict CS-AKI. However, NGAL levels were related to cardiopulmonary bypass (CPB) time and therefore may be a marker for overall perioperative pro-inflammatory insult (31).

**Biomarkers of hypoperfusion - H-FABP & VEGF**

H-FABP is a cytosolic protein found in myocardium (32) as well as skeletal muscle, brain and the kidney. H-FABP is a biomarker of myocardial injury that is more specific than myoglobin. H-FABP is released during myocardial ischemia at clinically detectable levels even prior to the advent of irreversible myocardial necrosis. H-FABP is superior to cardiac troponin I, creatine kinase-muscle/brain (CK-MB) and myoglobin for predicting all-cause mortality up to 5 years following cardiac surgery (32). In acute heart failure (AHF) patients, H-FABP levels were significantly higher in those who developed AKI when compared to control. The study concluded that serum H-FABP was a biomarker for AKI and a predictor of mortality in this patient group (33). Since H-FABP release reflects injured myocardium, reduced cardiac output may lead to hypoperfusion of vital organs including the kidney (33). H-FABP is elevated in postoperative renal failure in cardiac surgery in adults and in children (34,35).

VEGF is increased in acute hypoxia (hypoperfusion) (7), regulated by hypoxia-inducible factor-1α (HIF-1α) (36). In response to the hypoxia, VEGF is released to promote angiogenesis. Therefore, elevated levels of VEGF preoperatively may be caused by hypoxia suggesting that the body is already running in a state of relative hypoxia and the organs, including the kidney will already be compensating for this by instigating their control mechanisms. VEGF is beneficial in preventing AKI conversion to chronic renal dysfunction (37). In two groups of ICU patients: 102 with AKI and 102 non-AKI patients undergoing cardiac catheterization, urinary concentrations of VEGF were significantly higher in AKI patients than the control group (15). If VEGF prevents AKI progression to chronic renal failure (37), it is likely that the presence of significantly higher concentrations of VEGF in urine of AKI patients is an appropriate protective response.

**References**

1. Chatterjee PK, Hawksworth GM, McLay JS. Cytokine-stimulated nitric oxide production in the human renal proximal tubule and its modulation by natriuretic peptides: A novel immunomodulatory mechanism? Exp Nephrol. Switzerland; 1999;7:438–48.

2. McBride WT, Armstrong MA, Gilliland H, McMurray TJ. The balance of pro and anti-inflammatory cytokines in plasma and bronchoalveolar lavage (BAL) at paediatric cardiac surgery. Cytokine. England; 1996;8:724–9.

3. Bhargava R, Altmann CJ, Andres-Hernando A, Webb RG, Okamura K, Yang Y, et al. Acute lung injury and acute kidney injury are established by four hours in experimental sepsis and are improved with pre, but not post, sepsis administration of TNF-α antibodies. PLoS One. 2013;8:1–11.

4. de Fontnouvelle CA, Greenberg JH, Thiessen-Philbrook HR, Zappitelli M, Roth J, Kerr KF, et al. Interleukin-8 and Tumor Necrosis Factor Predict Acute Kidney Injury After Pediatric Cardiac Surgery. Ann Thorac Surg. 2017;104:2072–9.

5. Bihorac A, Baslanti TO, Cuenca AG, Hobson CE, Ang D, Efron PA, et al. Acute kidney injury is associated with early cytokine changes after trauma. J Trauma Acute Care Surg. United States; 2013;74:1005–13.

6. Kosugi T, Sato W. Midkine and the kidney: Health and diseases. Nephrol Dial Transplant. 2012;27:16–21.

7. Zhang R, Pan XH, Xiao L. Expression of vascular endothelial growth factor (VEGF) under hypoxia in placenta with intrahepatic cholestasis of pregnancy and its clinically pathological significance. Int J Clin Exp Pathol. 2015;8:11475–9.

8. Altara R, Manca M, Hessel MH, Gu Y, van Vark LC, Akkerhuis KM, et al. CXCL10 Is a Circulating Inflammatory Marker in Patients with Advanced Heart Failure: a Pilot Study. J Cardiovasc Transl Res. United States; 2016;9:302–14.

9. Merkle M, Ribeiro A, Koppel S, Wornle M. TNFalpha enhances TLR3-dependent effects on MMP-9 expression in human mesangial cells. Cell Biol Int. England; 2012;36:1155–60.

10. Merkle M, Ribeiro A, Wörnle M. TLR3-dependent regulation of cytokines in human mesangial cells: a novel role for IP-10 and TNF-α in hepatitis C-associated glomerulonephritis. Am J Physiol Physiol. 2011;301:F57–69.

11. Gorczyca D, Augustyniak D, Basiewicz-Worsztynowicz B, Karnas-Kalemba W. Serum and Urinary MIP-1α and IP-10 Levels in Children with Urinary Tract Infections*. Adv Clin Exp Med. 2015;23:933–8.

12. Arthur JM, Hill EG, Alge JL, Lewis EC, Neely BA, Janech MG, et al. Evaluation of 32 urine biomarkers to predict the progression of acute kidney injury after cardiac surgery. Kidney Int. Nature Publishing Group; 2014;85:431–8.

13. Bocci V, Pacini A, Pessina GP, Maioli E, Naldini A. Studies on tumor necrosis factor (TNF)-I. Pharmacokinetics of human recombinant TNF in rabbits and monkeys after intravenous administration. Gen Pharmacol. Pergamon; 1987;18:343–6.

14. Karakus S, Oktar T, Kucukgergin C, Kalelioglu I, Seckin S, Atar A, et al. Urinary IP-10, MCP-1, NGAL, Cystatin-C, and KIM-1 Levels in Prenatally Diagnosed Unilateral Hydronephrosis: The Search for an Ideal Biomarker. Urology. United States; 2016;87:185–92.

15. Vaidya VS, Waikar SS, Ferguson MA, Collings FB, Sunderland K, Gioules C, et al. Urinary Biomarkers for Sensitive and Specific Detection of Acute Kidney Injury in Humans. Clin Transl Sci. John Wiley & Sons, Ltd; 2008;1:200–8.

16. Peng H, Mao Y, Fu X, Feng Z, Xu J. Comparison of biomarkers in rat renal ischemia-reperfusion injury. Int J Clin Exp Med. E-Century Publishing Corporation; 2015;8:7577–84.

17. Basu RK, Donaworth E, Wheeler DS, Devarajan P, Wong HR. Antecedent acute kidney injury worsens subsequent endotoxin-induced lung inflammation in a two-hit mouse model. Am J Physiol Physiol. 2011;301:F597–604.

18. Maddens B, Vandendriessche B, Demon D, Vanholder R, Chiers K, Cauwels A, et al. Severity of sepsis-induced acute kidney injury in a novel mouse model is age dependent. Crit Care Med. United States; 2012;40:2638–46.

19. Munshi R, Johnson A, Siew ED, Ikizler TA, Ware LB, Wurfel MM, et al. MCP-1 Gene Activation Marks Acute Kidney Injury. J Am Soc Nephrol. 2011;22:165–75.

20. Rice JC, Spence JS, Yetman DL, Safirstein RL. Monocyte chemoattractant protein-1 expression correlates with monocyte infiltration in the post-ischemic kidney. Ren Fail. 2002;24:703–23.

21. Obermuller N, Geiger H, Weipert C, Urbschat A. Current developments in early diagnosis of acute kidney injury. Int Urol Nephrol. Netherlands; 2014;46:1–7.

22. Jastrow KM 3rd, Gonzalez EA, McGuire MF, Suliburk JW, Kozar RA, Iyengar S, et al. Early cytokine production risk stratifies trauma patients for multiple organ failure. J Am Coll Surg. United States; 2009;209:320–31.

23. Herbert F, Tchitchek N, Bansal D, Jacques J, Pathak S, Bécavin C, et al. Evidence of IL-17, IP-10, and IL-10 involvement in multiple-organ dysfunction and IL-17 pathway in acute renal failure associated to Plasmodium falciparum malaria. J Transl Med. BioMed Central; 2015;13:1–11.

24. Sato W, Kadomatsu K, Yuzawa Y, Muramatsu H, Hotta N, Matsuo S, et al. Midkine Is Involved in Neutrophil Infiltration into the Tubulointerstitium in Ischemic Renal Injury. J Immunol. 2001;167:3463–9.

25. Malyszko J, Koc-Zorawska E, Malyszko JS, Bachorzewska-Gajewska H, Dobrzycki S, Kobus G. Midkine: A novel and early biomarker of contrast-induced acute kidney injury in patients undergoing percutaneous coronary interventions. Biomed Res Int. 2015;2015.

26. Albert C, Albert A, Kube J, Bellomo R, Wettersten N, Kuppe H, et al. Urinary biomarkers may provide prognostic information for subclinical acute kidney injury after cardiac surgery. J Thorac Cardiovasc Surg. United States; 2018;155:2441-2452.e13.

27. Wang X, Wu T, Zhou F, Liu S, Zhou R, Zhu S, et al. IL12p40 regulates functional development of human CD4+ T cells: enlightenment by the elevated expressions of IL12p40 in patients with inflammatory bowel diseases. Medicine (Baltimore). United States; 2015;94:e613.

28. Ait-Lounis A, Laraba-Djebari F. TNF-alpha modulates adipose macrophage polarization to M1 phenotype in response to scorpion venom. Inflamm Res. Switzerland; 2015;64:929–36.

29. Cheung RYK, Lee JHS, Chan SSC, Liu DWT, Choy KW. A pilot study of urine cytokines in ketamine-associated lower urinary tract symptoms. Int Urogynecol J. England; 2014;25:1715–9.

30. Zhou F, Luo Q, Wang L, Han L. Diagnostic value of neutrophil gelatinase-associated lipocalin for early diagnosis of cardiac surgery-associated acute kidney injury: A meta-analysis. Eur J Cardio-thoracic Surg. 2016;49:746–55.

31. Garcia-Alvarez M, Glassford NJ, Betbese AJ, Ordonez J, Banos V, Argilaga M, et al. Urinary Neutrophil Gelatinase-Associated Lipocalin as Predictor of Short- or Long-Term Outcomes in Cardiac Surgery Patients. J Cardiothorac Vasc Anesth. United States; 2015;29:1480–8.

32. Muehlschlegel JD, Perry TE, Liu K-Y, Fox AA, Collard CD, Shernan SK, et al. Heart-type fatty acid binding protein is an independent predictor of death and ventricular dysfunction after coronary artery bypass graft surgery. Anesth Analg. NIH Public Access; 2010;111:1101–9.

33. Shirakabe A, Hata N, Kobayashi N, Okazaki H, Shinada T, Tomita K, et al. Serum Heart-Type Fatty Acid-Binding Protein Level Can Be Used to Detect Acute Kidney Injury on Admission and Predict an Adverse Outcome in Patients With Acute Heart Failure. Circ J. 2014;79:119–28.

34. Schaub JA, Garg AX, Coca SG, Testani JM, Shlipak MG, Eikelboom J, et al. Perioperative heart-type fatty acid binding protein is associated with acute kidney injury after cardiac surgery. Kidney Int. 2015;88:576–83.

35. Bucholz EM, Whitlock RP, Zappitelli M, Devarajan P, Eikelboom J, Garg AX, et al. Cardiac Biomarkers and Acute Kidney Injury After Cardiac Surgery. Pediatrics. 2015;135:e945–56.

36. Zhang Z, Dai H, Yu Y, Yang J, Hu C. Usefulness of heart-type fatty acid-binding protein in patients with severe sepsis. J Crit Care. United States; 2012;27:415.e13-8.

37. Rodriguez-Romo R, Benitez K, Barrera-Chimal J, Perez-Villalva R, Gomez A, Aguilar-Leon D, et al. AT1 receptor antagonism before ischemia prevents the transition of acute kidney injury to chronic kidney disease. Kidney Int. United States; 2016;89:363–73.
