# Supplementary material for: Blood and urinary cytokine balance and renal outcomes at cardiac surgery
Source: BMC Nephrol. 2021 Dec 8;22:406. doi: 10.1186/s12882-021-02621-6 (PMC8653550; doi:10.1186/s12882-021-02621-6)
Supplement: Supplementary file 2 — Additional file 2. Supplementary data, Tables 1–30. [file 12882_2021_2621_MOESM2_ESM.docx]

Supplemental Table 1. Blood pro-inflammatory and anti-inflammatory cytokines (Day 1)

| **Blood**  **cytokines** | **Pre-op**  **or**  **post-op** | **Day 1** | | | | |
| --- | --- | --- | --- | --- | --- | --- |
|  |  | **non-CS-AKI** | | **CS-AKI** | | **p value** |
|  |  | **n** | **median** | **n** | **median** |  |
| **Pro-inflammatory cytokines** | | | | | | |
| sIP-10 | pre-op | 289 | 114.27 | 25 | 156.80 | 0.014 |
| sIP-10 | post-op | 284 | 101.27 | 25 | 160.30 | 0.048 |
| sIL-12p40 | post-op | 284 | 286.39 | 25 | 385.52 | 0.039 |
| sMK | post-op | 243 | 1060.00 | 21 | 1905.26 | 0.050 |
| pIL-6 | pre-op | 283 | 2.20 | 25 | 3.54 | 0.003 |
| pMIP-1α | pre-op | 280 | 3.62 | 25 | 4.58 | 0.008 |
| pMIP-1α | post-op | 286 | 4.60 | 25 | 7.31 | 0.006 |
| pMCP-1 | pre-op | 283 | 130.00 | 25 | 142.00 | 0.032 |
| pMCP-1 | post-op | 289 | 203.00 | 25 | 299.00 | 0.009 |
| pNGAL | post op | 290 | 975.05 | 25 | 1303.85 | 0.019 |
| pTNFα | post-op | 289 | 2.36 | 25 | 3.14 | 0.037 |
| **Anti-inflammatory cytokines** | | | | | | |
| sTNFsr1 | pre-op | 289 | 0.35 | 25 | 0.50 | <0.001 |
| sTNFsr1 | post-op | 284 | 0.70 | 25 | 1.08 | <0.001 |
| sTNFsr2 | pre-op | 289 | 0.37 | 25 | 0.57 | 0.015 |
| sTNFsr2 | post-op | 284 | 0.73 | 25 | 1.26 | <0.001 |
| sIL-1RA | pre-op | 289 | 63.46 | 25 | 102.26 | 0.017 |
| sIL-1RA | post-op | 284 | 429.36 | 25 | 650.93 | 0.032 |

pre-op – preoperative, post-op – postoperative, CS-AKI – cardiac surgery acute kidney injury, n – number of patients, sIP-10 – serum interferon gamma - induced protein-10, sIL-12p40 – serum interleukin-12 subunit p40, sMK – serum midkine, pIL-6 – plasma interleukin-6, pMIP-1α – plasma macrophage inflammatory protein-1α, pMCP-1 – plasma monocyte chemotactic protein-1, pNGAL – plasma neutrophil gelatinase-associated lipocalin, pTNFα – plasma tumour necrosis factor α, sTNFsr1 – serum tumour necrosis factor soluble receptor 1, sTNFsr2 – serum tumour necrosis factor soluble receptor 2, sIL-1RA – serum interleukin-1 receptor antagonist

Supplemental Table 2. Urinary pro-inflammatory and anti-inflammatory cytokines (Day 1)

| **Urinary cytokines** | **Pre-op**  **or**  **post-op** | **Day 1** | | | | |
| --- | --- | --- | --- | --- | --- | --- |
|  |  | **non-CS-AKI** | | **CS-AKI** | | **p value** |
|  |  | **n** | **median** | **n** | **median** |  |
| **Pro-inflammatory cytokines** | | | | | | |
| uIP-10 | post-op | 286 | 13.12 | 25 | 25.99 | 0.001 |
| uNGAL | post-op | 276 | 144.01 | 25 | 227.39 | 0.001 |
| **Anti-inflammatory cytokines** | | | | | | |
| uTNFsr2 | post-op | 285 | 8.18 | 25 | 9.75 | 0.027 |

pre-op – preoperative, post-op – postoperative, CS-AKI – cardiac surgery acute kidney injury, n – number of patients, uIP-10 – urinary interferon gamma - induced protein-10, uNGAL – urinary neutrophil gelatinase-associated lipocalin, uTNFsr2 – urinary tumour necrosis factor soluble receptor 2

Supplemental Table 3. Blood pro-inflammatory and anti-inflammatory cytokines (Day 2)

| **Blood**  **cytokines** | **Pre-op**  **or**  **post-op** | **Day 2** | | | | |
| --- | --- | --- | --- | --- | --- | --- |
|  |  | **non-CS-AKI** | | **CS-AKI** | | **p value** |
|  |  | **n** | **median** | **n** | **median** |  |
| **Pro-inflammatory cytokines** | | | | | | |
| sIP-10 | pre-op | 275 | 114.50 | 55 | 137.14 | 0.043 |
| sIL-12p40 | pre-op | 275 | 371.19 | 55 | 539.87 | <0.001 |
| sIL-12p40 | post-op | 269 | 273.87 | 56 | 438.05 | <0.001 |
| sMK | post-op | 224 | 990.10 | 50 | 2412.33 | <0.001 |
| pIL-6 | pre-op | 269 | 2.24 | 55 | 2.92 | 0.007 |
| pIL-8 | post-op | 274 | 9.01 | 56 | 11.44 | 0.036 |
| pMIP-1α | pre-op | 266 | 3.61 | 55 | 4.45 | 0.012 |
| pMIP-1α | post-op | 271 | 4.52 | 56 | 6.41 | <0.001 |
| pMCP-1 | pre-op | 269 | 130.00 | 55 | 140.00 | 0.034 |
| pMCP-1 | post-op | 274 | 198.00 | 56 | 234.50 | 0.006 |
| pNGAL | pre-op | 270 | 573.54 | 55 | 672.83 | 0.002 |
| pNGAL | post op | 275 | 964.87 | 56 | 1463.26 | <0.001 |
| pTNFα | pre-op | 269 | 2.06 | 55 | 2.55 | 0.001 |
| pTNFα | post-op | 274 | 2.34 | 56 | 3.04 | 0.001 |
| **Anti-inflammatory cytokines** | | | | | | |
| sTNFsr1 | pre-op | 275 | 0.33 | 55 | 0.54 | <0.001 |
| sTNFsr1 | post-op | 269 | 0.69 | 56 | 1.03 | <0.001 |
| sTNFsr2 | pre-op | 275 | 0.36 | 55 | 0.57 | <0.001 |
| sTNFsr2 | post-op | 269 | 0.73 | 56 | 1.20 | <0.001 |
| sIL-1RA | pre-op | 275 | 62.37 | 55 | 84.29 | 0.015 |
| sIL-1RA | post-op | 269 | 414.26 | 56 | 701.17 | <0.001 |

pre-op – preoperative, post-op – postoperative, CS-AKI – cardiac surgery acute kidney injury, n – number of patients, sIP-10 – serum interferon gamma - induced protein-10, sIL-12p40 – serum interleukin-12 subunit p40, sMK – serum midkine, pIL-6 – plasma interleukin-6, pIL-8 – plasma interleukin-8, pMIP-1α – plasma macrophage inflammatory protein-1α, pMCP-1 – plasma monocyte chemotactic protein-1, pNGAL – plasma neutrophil gelatinase-associated lipocalin, pTNFα – plasma tumour necrosis factor α, sTNFsr1 – serum tumour necrosis factor soluble receptor 1, sTNFsr2 – serum tumour necrosis factor soluble receptor 2, sIL-1RA – serum interleukin-1 receptor antagonist

Supplemental Table 4. Urinary pro-inflammatory and anti-inflammatory cytokines (Day 2)

| **Urinary cytokines** | **Pre-op**  **or**  **post-op** | **Day 2** | | | | |
| --- | --- | --- | --- | --- | --- | --- |
|  |  | **non-CS-AKI** | | **CS-AKI** | | **p value** |
|  |  | **n** | **median** | **n** | **median** |  |
| **Pro-inflammatory cytokines** | | | | | | |
| uIP-10 | pre-op | 273 | 5.50 | 56 | 9.42 | 0.031 |
| uIP-10 | post-op | 271 | 13.51 | 56 | 21.08 | 0.012 |
| uIL-12p40 | pre-op | 273 | 1.99 | 56 | 2.66 | 0.028 |
| uIL-12p40 | post-op | 271 | 2.90 | 56 | 4.82 | <0.001 |
| uNGAL | post-op | 262 | 141.19 | 55 | 230.31 | 0.002 |
| **Anti-inflammatory cytokines** | | | | | | |
| uTNFsr1 | pre-op | 272 | 0.52 | 56 | 0.71 | 0.026 |

pre-op – preoperative, post-op – postoperative, CS-AKI – cardiac surgery acute kidney injury, n – number of patients, uIP-10 – urinary interferon gamma - induced protein-10, uIL-12p40 – urinary interleukin-12 subunit p40, uNGAL – urinary neutrophil gelatinase-associated lipocalin, uTNFsr1 – urinary tumour necrosis factor soluble receptor 1

Supplemental Table 5. Blood pro-inflammatory and anti-inflammatory cytokines (Day 5)

| **Blood**  **cytokines** | **Pre-op**  **or**  **post-op** | **Day 5** | | | | |
| --- | --- | --- | --- | --- | --- | --- |
|  |  | **non-CS-AKI** | | **CS-AKI** | | **p value** |
|  |  | **n** | **median** | **n** | **median** |  |
| **Pro-inflammatory cytokines** | | | | | | |
| sIL-12p40 | pre-op | 298 | 388.51 | 22 | 712.18 | <0.001 |
| sIL-12p40 | post-op | 293 | 293.16 | 22 | 444.80 | 0.003 |
| sMK | post-op | 248 | 1063.82 | 18 | 2651.94 | 0.010 |
| pIL-6 | pre-op | 292 | 2.24 | 22 | 3.05 | 0.030 |
| pIL-8 | post-op | 298 | 8.99 | 22 | 14.57 | 0.010 |
| pMIP-1α | pre-op | 289 | 3.62 | 22 | 4.71 | 0.002 |
| pMIP-1α | post-op | 295 | 4.79 | 22 | 7.30 | 0.003 |
| pMCP-1 | pre-op | 292 | 129.55 | 22 | 154.50 | 0.017 |
| pMCP-1 | post-op | 298 | 202.50 | 22 | 255.57 | 0.026 |
| pNGAL | pre-op | 293 | 580.62 | 22 | 832.09 | 0.004 |
| pNGAL | post op | 299 | 978.72 | 22 | 1509.05 | 0.001 |
| pTNFα | pre-op | 292 | 2.06 | 22 | 2.66 | 0.002 |
| pTNFα | post-op | 298 | 2.37 | 22 | 3.15 | 0.008 |
| **Anti-inflammatory cytokines** | | | | | | |
| sTNFsr1 | pre-op | 298 | 0.35 | 22 | 0.56 | <0.001 |
| sTNFsr1 | post-op | 293 | 0.70 | 22 | 1.10 | <0.001 |
| sTNFsr2 | pre-op | 298 | 0.37 | 22 | 0.55 | <0.001 |
| sTNFsr2 | post-op | 293 | 0.74 | 22 | 1.26 | <0.001 |
| sIL-1RA | post-op | 293 | 430.69 | 22 | 1066.10 | 0.001 |

pre-op – preoperative, post-op – postoperative, CS-AKI – cardiac surgery acute kidney injury, n – number of patients, sIL-12p40 – serum interleukin-12 subunit p40, sMK – serum midkine, pIL-6 – plasma interleukin-6, pIL-8 – plasma interleukin 8, pMIP-1α – plasma macrophage inflammatory protein-1α, pMCP-1 – plasma monocyte chemotactic protein 1, pNGAL – plasma neutrophil gelatinase-associated lipocalin, pTNFα – plasma tumour necrosis factor α, sTNFsr1 – serum tumour necrosis factor soluble receptor 1, sTNFsr2 – serum tumour necrosis factor soluble receptor 2, sIL-1RA – serum interleukin -1 receptor antagonist

Supplemental Table 6. Urinary pro-inflammatory cytokines (Day 5)

| **Urinary cytokines** | **Pre-op**  **or**  **post-op** | **Day 5** | | | | |
| --- | --- | --- | --- | --- | --- | --- |
|  |  | **non-CS-AKI** | | **CS-AKI** | | **p value** |
|  |  | **n** | **median** | **n** | **median** |  |
| **Pro-inflammatory cytokines** | | | | | | |
| uIP-10 | post-op | 295 | 13.23 | 22 | 26.55 | 0.019 |
| uIL-12p40 | pre-op | 297 | 1.88 | 22 | 3.13 | 0.003 |
| uIL-12p40 | post-op | 295 | 3.07 | 22 | 4.50 | 0.040 |
| uNGAL | post-op | 286 | 145.89 | 21 | 241.82 | 0.010 |

pre-op – preoperative, post-op – postoperative, CS-AKI –cardiac surgery acute kidney injury, n – number of patients, uIP-10 – urinary interferon gamma - induced protein-10, uIL-12p40 – urinary interleukin-12 subunit p40, uNGAL – urinary neutrophil gelatinase-associated lipocalin

Supplemental Table 7. Ratios of blood anti-inflammatory/blood pro-inflammatory cytokines (Day 1)

| **Blood anti-inflammatory/ blood pro-inflammatory cytokines** | **Pre-op**  **or**  **post-op** | **Day 1** | | | | |
| --- | --- | --- | --- | --- | --- | --- |
|  |  | **non-CS-AKI** | | **CS-AKI** | | **p value** |
|  |  | **n** | **ratio** | **n** | **ratio** |  |
| sTNFsr1/ pTNFα | pre-op | 237 | 0.158 | 23 | 0.207 | 0.005 |
| sTNFsr1/ pTNFα | post-op | 240 | 0.272 | 23 | 0.326 | 0.038 |
| sTNFsr1/ pIL-6 | post-op | 280 | 0.006 | 25 | 0.011 | 0.014 |
| sTNFsr1/sIL-12p40 | post-op | 284 | 0.002 | 25 | 0.003 | 0.046 |
| sTNFsr1/ pMIP-1α | pre-op | 278 | 0.099 | 25 | 0.121 | 0.028 |
| sTNFsr1/ pMCP-1 | pre-op | 282 | 0.0028 | 25 | 0.0034 | 0.037 |
| sTNFsr1/ pNGAL | pre-op | 283 | 0.0006 | 25 | 0.0008 | 0.001 |
| sTNFsr1/ pNGAL | post-op | 282 | 0.0007 | 25 | 0.0009 | 0.014 |
| sTNFsr2/ pIL-6 | post-op | 280 | 0.006 | 25 | 0.012 | 0.015 |
| sTNFsr2/ sIL-12p40 | post-op | 284 | 0.003 | 25 | 0.004 | 0.011 |
| sTNFsr2/ pNGAL | pre-op | 283 | 0.0007 | 25 | 0.0009 | 0.015 |
| sTNFsr2/ pNGAL | post-op | 282 | 0.0008 | 25 | 0.0011 | 0.023 |
| sIL-1RA/ pIL-6 | post-op | 280 | 3.789 | 25 | 5.657 | 0.047 |
| sIL-1RA/ pNGAL | pre-op | 283 | 0.115 | 25 | 0.162 | 0.031 |

pre-op – preoperative, post-op – postoperative, CS-AKI – cardiac surgery acute kidney injury, n – number of patients, sTNFsr1 – serum tumour necrosis factor soluble receptor 1, pTNFα – plasma tumour necrosis factor α, pIL-6 – plasma interleukin-6, sIL-12p40 – serum interleukin 12 subunit p40, pMIP-1α – plasma macrophage inflammatory protein 1α, pMCP-1 – plasma monocyte chemotactic protein 1, pNGAL – plasma neutrophil gelatinase-associated lipocalin, sTNFsr2 – serum tumour necrosis factor soluble receptor 2, sIL-1RA – serum interleukin 1 receptor antagonist

Supplemental Table 8. Ratios of urinary anti-inflammatory/urinary pro-inflammatory cytokines (Day 1)

| **Urinary anti-inflammatory/ urinary pro-inflammatory cytokines** | **Pre-op**  **or**  **post-op** | **Day 1** | | | | |
| --- | --- | --- | --- | --- | --- | --- |
|  |  | **non-CS-AKI** | | **CS-AKI** | | **p value** |
|  |  | **n** | **ratio** | **n** | **ratio** |  |
| uIL-1RA/ uIP-10 | post-op | 266 | 0.408 | 25 | 0.268 | 0.013 |
| uIL-1RA/ uNGAL | post-op | 274 | 0.045 | 25 | 0.037 | 0.017 |

pre-op – preoperative, post-op – postoperative, CS-AKI – cardiac surgery acute kidney injury, n – number of patients, uIL-1RA – urinary interleukin-1 receptor antagonist, uIP-10 – urinary interferon gamma - induced protein-10, uNGAL – urinary neutrophil gelatinase-associated lipocalin

Supplemental Table 9. Ratios of urinary anti-inflammatory/blood pro-inflammatory cytokines (Day 1)

| **Urinary anti-inflammatory/ blood pro-inflammatory cytokines** | **Pre-op**  **or**  **post-op** | **Day 1** | | | | |
| --- | --- | --- | --- | --- | --- | --- |
|  |  | **non-CS-AKI** | | **CS-AKI** | | **p value** |
|  |  | **n** | **ratio** | **n** | **ratio** |  |
| uIL-1RA/ pTNFα | post-op | 242 | 712.086 | 23 | 556.637 | 0.014 |
| uIL-1RA/ pIL-6 | pre-op | 262 | 552.969 | 25 | 358.745 | 0.028 |
| uIL-1RA/ pIL-8 | post-op | 282 | 209.435 | 25 | 150.119 | 0.021 |
| uIL-1RA/ sIP-10 | post-op | 279 | 18.212 | 25 | 11.138 | 0.019 |
| uIL-1RA/ sIL-12p40 | post-op | 279 | 6.463 | 25 | 4.350 | 0.007 |
| uIL-1RA/ pMIP-1α | post-op | 278 | 389.484 | 25 | 218.909 | <0.001 |
| uIL-1RA/ pMCP-1 | post-op | 282 | 9.029 | 25 | 5.407 | 0.001 |
| uIL-1RA/ sMK | post-op | 237 | 1.774 | 21 | 0.955 | 0.026 |
| uIL-1RA/ pNGAL | post-op | 282 | 1.834 | 25 | 1.241 | 0.003 |

pre-op – preoperative, post-op – postoperative, CS-AKI – cardiac surgery acute kidney injury, n – number of patients, uIL-1RA – urinary interleukin-1 receptor antagonist, pTNFα – plasma tumour necrosis factor α, pIL-6 – plasma interleukin-6, pIL-8 – plasma interleukin 8, sIP-10 – serum interferon gamma - induced protein-10, sIL-12p40 – serum interleukin 12 subunit p40, pMIP-1α – plasma macrophage inflammatory protein 1α, pMCP-1 – plasma monocyte chemotactic protein 1, sMK – serum midkine, pNGAL – plasma neutrophil gelatinase-associated lipocalin

Supplemental Table 10. Blood hypoperfusion biomarkers (Day 1)

| **Blood hypoperfusion biomarkers** | **Pre-op**  **or**  **post-op** | **Day 1** | | | | |
| --- | --- | --- | --- | --- | --- | --- |
|  |  | **non-CS-AKI** | | **CS-AKI** | | **p value** |
|  |  | **n** | **median** | **n** | **median** |  |
| **Serum biomarker** | | | | | | |
| sH-FABP | pre-op | 288 | 4.935 | 24 | 7.110 | 0.021 |
| sH-FABP | post-op | 287 | 19.500 | 25 | 46.780 | <0.001 |
| **Plasma biomarker** | | | | | | |
| pVEGF | pre-op | 283 | 31.000 | 25 | 37.000 | 0.022 |

pre-op – preoperative, post-op – postoperative, CS-AKI – cardiac surgery acute kidney injury, n – number of patients, sH-FABP – serum heart-type fatty acid-binding protein, pVEGF – plasma vascular endothelial growth factor

Supplemental Table 11. Ratios of urinary anti-inflammatory/blood hypoperfusion biomarkers (Day 1)

| **Urinary anti-inflammatory/ blood hypoperfusion biomarkers** | **Pre-op**  **or**  **post-op** | **Day 1** | | | | |
| --- | --- | --- | --- | --- | --- | --- |
|  |  | **non-CS-AKI** | | **CS-AKI** | | **p value** |
|  |  | **n** | **ratio** | **n** | **ratio** |  |
| uTNFsr1/ sH-FABP | post-op | 278 | 0.323 | 25 | 0.140 | 0.001 |
| uTNFsr2/ sH-FABP | post-op | 279 | 0.402 | 25 | 0.149 | 0.001 |
| uIL-1RA/ sH-FABP | post-op | 270 | 3.059 | 25 | 2.566 | <0.001 |

pre-op – preoperative, post-op – postoperative, CS-AKI – cardiac surgery acute kidney injury, n – number of patients, uTNFsr1 – urinary tumour necrosis factor soluble receptor 1, sH-FABP – serum heart-type fatty acid-binding protein, uTNFsr2 – urinary tumour necrosis factor soluble receptor 2, uIL-1RA – urinary interleukin-1 receptor antagonist

Supplemental Table 12. Ratios of blood anti-inflammatory/urinary anti-inflammatory cytokines (Day 1)

| **Blood anti-inflammatory/ urinary anti-inflammatory cytokines** | **Pre-op**  **or**  **post-op** | **Day 1** | | | | |
| --- | --- | --- | --- | --- | --- | --- |
|  |  | **non-CS-AKI** | | **CS-AKI** | | **p value** |
|  |  | **n** | **ratio** | **n** | **ratio** |  |
| sTNFsr1/ uTNFsr1 | post-op | 277 | 0.109 | 25 | 0.136 | 0.029 |
| sTNFsr1/ uTNFsr2 | post-op | 278 | 0.088 | 25 | 0.121 | 0.021 |
| sTNFsr1/ uIL-1RA | pre-op | 285 | 0.0002 | 25 | 0.0003 | 0.026 |
| sTNFsr1/ uIL-1RA | post-op | 279 | 0.0004 | 25 | 0.0006 | <0.001 |
| sTNFsr2/ uTNFsr1 | post-op | 277 | 0.119 | 25 | 0.157 | 0.020 |
| sTNFsr2/ uTNFsr2 | post-op | 278 | 0.099 | 25 | 0.141 | 0.018 |
| sTNFsr2/ uIL-1RA | post-op | 279 | 0.0004 | 25 | 0.0007 | <0.001 |
| sIL-1RA/ uIL-1RA | post-op | 279 | 0.236 | 25 | 0.489 | 0.006 |

pre-op – preoperative, post-op – postoperative, CS-AKI – cardiac surgery acute kidney injury, n – number of patients, sTNFsr1 – serum tumour necrosis factor soluble receptor 1, uTNFsr1 – urinary tumour necrosis factor soluble receptor 1, uTNFsr2 – urinary tumour necrosis factor soluble receptor 2, uIL-1RA – urinary interleukin-1 receptor antagonist, sTNFsr2 – serum tumour necrosis factor soluble receptor 2, sIL-1RA – serum interleukin-1 receptor antagonist

Supplemental Table 13. Ratios of blood anti-inflammatory/blood pro-inflammatory cytokines (Day 2)

| **Blood anti-inflammatory/ blood pro-inflammatory cytokines** | **Pre-op**  **or**  **post-op** | **Day 2** | | | | |
| --- | --- | --- | --- | --- | --- | --- |
|  |  | **non-CS-AKI** | | **CS-AKI** | | **p value** |
|  |  | **n** | **ratio** | **n** | **ratio** |  |
| sTNFsr1/ pTNFα | pre-op | 222 | 0.156 | 51 | 0.201 | 0.001 |
| sTNFsr1/ pTNFα | post-op | 226 | 0.266 | 52 | 0.327 | 0.002 |
| sTNFsr1/ pIL-6 | post-op | 265 | 0.006 | 56 | 0.007 | 0.010 |
| sTNFsr1/ pIL-8 | pre-op | 268 | 0.124 | 55 | 0.150 | 0.007 |
| sTNFsr1/ sIP-10 | pre-op | 275 | 0.003 | 55 | 0.004 | 0.008 |
| sTNFsr1/ pMIP-1α | pre-op | 265 | 0.098 | 54 | 0.127 | <0.001 |
| sTNFsr1/ pMCP-1 | pre-op | 268 | 0.003 | 55 | 0.004 | <0.001 |
| sTNFsr1/ pMCP-1 | post-op | 266 | 0.003 | 56 | 0.004 | 0.003 |
| sTNFsr1/ sMK | pre-op | 222 | 0.0006 | 50 | 0.0008 | 0.047 |
| sTNFsr1/ sMK | post-op | 221 | 0.0007 | 50 | 0.0005 | 0.033 |
| sTNFsr1/ pNGAL | pre-op | 269 | 0.0006 | 55 | 0.0008 | <0.001 |
| sTNFsr2/ pTNFα | pre-op | 222 | 0.154 | 51 | 0.211 | 0.002 |
| sTNFsr2/ pTNFα | post-op | 226 | 0.294 | 52 | 0.365 | 0.003 |
| sTNFsr2/ pIL-6 | post-op | 265 | 0.006 | 56 | 0.009 | 0.002 |
| sTNFsr2/ pIL-8 | pre-op | 268 | 0.118 | 55 | 0.168 | 0.004 |
| sTNFsr2/ pIL-8 | post-op | 266 | 0.082 | 56 | 0.101 | 0.038 |
| sTNFsr2/ sIP-10 | pre-op | 275 | 0.003 | 55 | 0.004 | 0.016 |
| sTNFsr2/ sIP-10 | post-op | 269 | 0.007 | 56 | 0.009 | 0.028 |
| sTNFsr2/ pMIP-1α | pre-op | 265 | 0.100 | 54 | 0.128 | 0.002 |
| sTNFsr2/ pMCP-1 | pre-op | 268 | 0.003 | 55 | 0.004 | <0.001 |
| sTNFsr2/ pMCP-1 | post-op | 266 | 0.004 | 56 | 0.005 | 0.001 |
| sTNFsr2/ sMK | pre-op | 222 | 0.0006 | 50 | 0.0010 | 0.008 |
| sTNFsr2/ pNGAL | pre-op | 269 | 0.0007 | 55 | 0.0009 | 0.001 |
| sIL-1RA/ pTNFα | post-op | 226 | 176.803 | 52 | 241.395 | 0.010 |
| sIL-1RA/ pIL-6 | post-op | 265 | 3.711 | 56 | 5.605 | 0.003 |
| sIL-1RA/ sIP-10 | post-op | 269 | 4.124 | 56 | 5.717 | 0.050 |
| sIL-1RA/ pMCP-1 | post-op | 266 | 2.111 | 56 | 3.036 | 0.009 |

pre-op – preoperative, post-op – postoperative, CS-AKI – cardiac surgery acute kidney injury, n – number of patients, sTNFsr1 – serum tumour necrosis factor soluble receptor 1, pTNFα – plasma tumour necrosis factor α, pIL-6 – plasma interleukin-6, pIL-8 – plasma interleukin 8, sIP-10 – serum interferon gamma - induced protein-10, pMIP-1α – plasma macrophage inflammatory protein 1α, pMCP-1 – plasma monocyte chemotactic protein 1, sMK – serum midkine, pNGAL – plasma neutrophil gelatinase-associated lipocalin, sTNFsr2 – serum tumour necrosis factor soluble receptor 2, sIL-1RA – serum interleukin 1 receptor antagonist

Supplemental Table 14. Ratios of urinary anti-inflammatory/urinary pro-inflammatory cytokines (Day 2)

| **Urinary anti-inflammatory/ urinary pro-inflammatory cytokines** | **Pre-op**  **or**  **post-op** | **Day 2** | | | | |
| --- | --- | --- | --- | --- | --- | --- |
|  |  | **non-CS-AKI** | | **CS-AKI** | | **p value** |
|  |  | **n** | **ratio** | **n** | **ratio** |  |
| uTNFsr2/ uIP-10 | post-op | 253 | 0.477 | 54 | 0.331 | 0.013 |
| uTNFsr2/ uNGAL | post-op | 261 | 0.052 | 55 | 0.040 | 0.008 |
| uIL-1RA/ uIP-10 | post-op | 253 | 121.742 | 54 | 87.261 | 0.013 |
| uIL-1RA/ uIL-12p40 | post-op | 180 | 504.560 | 48 | 350.832 | 0.005 |
| uIL-1RA/ uNGAL | post-op | 261 | 13.150 | 55 | 8.193 | 0.002 |

pre-op – preoperative, post-op – postoperative, CS-AKI – cardiac surgery acute kidney injury, n – number of patients, uTNFsr2 – urinary tumour necrosis factor soluble receptor 2, uIP-10 – urinary interferon gamma - induced protein-10, uNGAL – urinary neutrophil gelatinase-associated lipocalin, uIL-1RA – urinary interleukin-1 receptor antagonist, uIL-12p40 – urinary interleukin-12 subunit p40

Supplemental Table 15. Ratios of urinary anti-inflammatory/blood pro-inflammatory cytokines (Day 2)

| **Urinary anti-inflammatory/ blood pro-inflammatory cytokines** | **Pre-op**  **or**  **post-op** | **Day 2** | | | | |
| --- | --- | --- | --- | --- | --- | --- |
|  |  | **non-CS-AKI** | | **CS-AKI** | | **p value** |
|  |  | **n** | **ratio** | **n** | **ratio** |  |
| uTNFsr1/ sIL-12p40 | post-op | 262 | 0.023 | 56 | 0.019 | 0.035 |
| uTNFsr1/ sMK | post-op | 216 | 0.006 | 50 | 0.003 | 0.002 |
| uTNFsr1/ pNGAL | post-op | 265 | 0.007 | 56 | 0.006 | 0.047 |
| uTNFsr2/ sIL-12p40 | post-op | 263 | 0.029 | 56 | 0.022 | 0.002 |
| uTNFsr2/ pMIP-1α | post-op | 262 | 1.633 | 56 | 1.448 | 0.044 |
| uTNFsr2/ sMK | post-op | 217 | 0.008 | 50 | 0.004 | <0.001 |
| uTNFsr2/ pNGAL | post-op | 266 | 0.008 | 56 | 0.006 | 0.003 |
| uIL-1RA/ pTNFα | post-op | 228 | 724.509 | 52 | 588.911 | 0.030 |
| uIL-1RA/ pIL-6 | pre-op | 248 | 570.518 | 55 | 419.013 | 0.030 |
| uIL-1RA/ pIL-8 | post-op | 267 | 208.048 | 56 | 163.944 | 0.020 |
| uIL-1RA/ sIL-12p40 | pre-op | 271 | 3.558 | 55 | 2.969 | 0.004 |
| uIL-1RA/ sIL-12p40 | post-op | 264 | 6.786 | 56 | 4.227 | <0.001 |
| uIL-1RA/ pMIP-1α | post-op | 263 | 398.101 | 56 | 284.401 | <0.001 |
| uIL-1RA/ pMCP-1 | post-op | 267 | 9.125 | 56 | 7.336 | 0.003 |
| uIL-1RA/ sMK | post-op | 218 | 1.876 | 50 | 0.772 | <0.001 |
| uIL-1RA/ pNGAL | pre-op | 265 | 2.606 | 55 | 2.063 | 0.023 |
| uIL-1RA/ pNGAL | post-op | 267 | 1.928 | 56 | 1.163 | <0.001 |

pre-op – preoperative, post-op – postoperative, CS-AKI – cardiac surgery acute kidney injury, n – number of patients, uTNFsr1 – urinary tumour necrosis factor soluble receptor 1, sIL-12p40 – serum interleukin 12 subunit p40, sMK – serum midkine, pNGAL – plasma neutrophil gelatinase-associated lipocalin, uTNFsr2 – urinary tumour necrosis factor soluble receptor 2, pMIP-1α – plasma macrophage inflammatory protein 1α, uIL-1RA – urinary interleukin-1 receptor antagonist, pTNFα – plasma tumour necrosis factor α, pIL-6 – plasma interleukin-6, pIL-8 – plasma interleukin 8, pMCP-1 – plasma monocyte chemotactic protein 1

Supplemental Table 16. Blood hypoperfusion biomarkers (Day 2)

| **Blood hypoperfusion biomarkers** | **Pre-op**  **or**  **post-op** | **Day 2** | | | | |
| --- | --- | --- | --- | --- | --- | --- |
|  |  | **non-CS-AKI** | | **CS-AKI** | | **p value** |
|  |  | **n** | **median** | **n** | **median** |  |
| **Serum biomarker** | | | | | | |
| sH-FABP | pre-op | 274 | 4.880 | 54 | 6.430 | 0.004 |
| sH-FABP | post-op | 272 | 18.305 | 56 | 44.760 | <0.001 |
| **Plasma biomarker** | | | | | | |
| pVEGF | pre-op | 269 | 30.000 | 55 | 34.070 | 0.005 |

pre-op – preoperative, post-op – postoperative, CS-AKI – cardiac surgery acute kidney injury, n – number of patients, sH-FABP – serum heart-type fatty acid-binding protein, pVEGF – plasma vascular endothelial growth factor

Supplemental Table 17. Ratios of urinary anti-inflammatory/blood hypoperfusion biomarkers (Day 2)

| **Urinary anti-inflammatory/ blood hypoperfusion biomarkers** | **Pre-op**  **or**  **post-op** | **Day 2** | | | | |
| --- | --- | --- | --- | --- | --- | --- |
|  |  | **non-CS-AKI** | | **CS-AKI** | | **p value** |
|  |  | **n** | **ratio** | **n** | **ratio** |  |
| uTNFsr1/ sH-FABP | post-op | 263 | 0.323 | 56 | 0.156 | <0.001 |
| uTNFsr2/ sH-FABP | post-op | 264 | 0.434 | 56 | 0.182 | <0.001 |
| uIL-1RA/ sH-FABP | pre-op | 257 | 4.399 | 53 | 3.916 | 0.050 |
| uIL-1RA/ sH-FABP | post-op | 256 | 3.104 | 55 | 2.529 | <0.001 |

pre-op – preoperative, post-op – postoperative, CS-AKI – cardiac surgery acute kidney injury, n – number of patients, uTNFsr1 – urinary tumour necrosis factor soluble receptor 1, sH-FABP – serum heart-type fatty acid-binding protein, uTNFsr2 – urinary tumour necrosis factor soluble receptor 2, uIL-1RA – urinary interleukin-1 receptor antagonist

Supplemental Table 18. Ratios of blood anti-inflammatory/urinary anti-inflammatory cytokines (Day 2)

| **Blood anti-inflammatory/ urinary anti-inflammatory cytokines** | **Pre-op**  **or**  **post-op** | **Day 2** | | | | |
| --- | --- | --- | --- | --- | --- | --- |
|  |  | **non-CS-AKI** | | **CS-AKI** | | **p value** |
|  |  | **n** | **ratio** | **n** | **ratio** |  |
| sTNFsr1/ uTNFsr1 | post-op | 262 | 0.108 | 56 | 0.123 | 0.044 |
| sTNFsr1/ uTNFsr2 | post-op | 263 | 0.087 | 56 | 0.117 | 0.003 |
| sTNFsr1/ uIL-1RA | pre-op | 271 | 0.0002 | 55 | 0.0003 | <0.001 |
| sTNFsr1/ uIL-1RA | post-op | 264 | 0.0004 | 56 | 0.0006 | <0.001 |
| sTNFsr2/ uTNFsr1 | post-op | 262 | 0.118 | 56 | 0.154 | 0.011 |
| sTNFsr2/ uTNFsr2 | post-op | 263 | 0.098 | 56 | 0.137 | <0.001 |
| sTNFsr2/ uIL-1RA | pre-op | 271 | 0.0002 | 55 | 0.0004 | <0.001 |
| sTNFsr2/ uIL-1RA | post-op | 264 | 0.0004 | 56 | 0.0007 | <0.001 |
| sIL-1RA/ uTNFsr2 | post-op | 263 | 56.157 | 56 | 87.080 | 0.011 |
| sIL-1RA/ uIL-1RA | post-op | 264 | 0.232 | 56 | 0.442 | <0.001 |

pre-op – preoperative, post-op – postoperative, CS-AKI – cardiac surgery acute kidney injury, n – number of patients, sTNFsr1 – serum tumour necrosis factor soluble receptor 1, uTNFsr1 – urinary tumour necrosis factor soluble receptor 1, uTNFsr2 – urinary tumour necrosis factor soluble receptor 2, uIL-1RA – urinary interleukin-1 receptor antagonist, sTNFsr2 – serum tumour necrosis factor soluble receptor 2, sIL-1RA – serum interleukin-1 receptor antagonist

Supplemental Table 19. Ratios of blood anti-inflammatory/blood pro-inflammatory cytokines (Day 5)

| **Blood anti-inflammatory/ blood pro-inflammatory cytokines** | **Pre-op**  **or**  **post-op** | **Day 5** | | | | |
| --- | --- | --- | --- | --- | --- | --- |
|  |  | **non-CS-AKI** | | **CS-AKI** | | **p value** |
|  |  | **n** | **ratio** | **n** | **ratio** |  |
| sTNFsr1/ pTNFα | pre-op | 243 | 0.162 | 21 | 0.184 | 0.044 |
| sTNFsr1/ pIL-6 | post-op | 289 | 0.006 | 22 | 0.009 | 0.037 |
| sTNFsr1/ sIP-10 | pre-op | 298 | 0.003 | 22 | 0.004 | 0.021 |
| sTNFsr1/ pMIP-1α | pre-op | 287 | 0.101 | 22 | 0.129 | 0.011 |
| sTNFsr1/ pMCP-1 | pre-op | 291 | 0.003 | 22 | 0.004 | 0.002 |
| sTNFsr1/ pNGAL | pre-op | 292 | 0.0006 | 22 | 0.0008 | 0.061 |
| sTNFsr2/ pTNFα | pre-op | 243 | 0.167 | 21 | 0.225 | 0.012 |
| sTNFsr2/ pIL-6 | post-op | 289 | 0.006 | 22 | 0.010 | 0.039 |
| sTNFsr2/ pIL-8 | pre-op | 291 | 0.127 | 22 | 0.182 | 0.028 |
| sTNFsr2/ sIP-10 | pre-op | 298 | 0.003 | 22 | 0.005 | 0.019 |
| sTNFsr2/ pMIP-1α | pre-op | 287 | 0.106 | 22 | 0.177 | 0.008 |
| sTNFsr2/ pMCP-1 | pre-op | 291 | 0.003 | 22 | 0.004 | 0.003 |
| sTNFsr2/ pNGAL | pre-op | 292 | 0.0007 | 22 | 0.0009 | 0.039 |
| sIL-1RA/ pTNFα | post-op | 247 | 174.979 | 21 | 321.413 | 0.009 |
| sIL-1RA/ pIL-6 | post-op | 289 | 3.853 | 22 | 6.738 | 0.004 |
| sIL-1RA/ pMCP-1 | post-op | 290 | 2.122 | 22 | 3.414 | 0.016 |

pre-op – preoperative, post-op – postoperative, CS-AKI – cardiac surgery acute kidney injury, n – number of patients, sTNFsr1 – serum tumour necrosis factor soluble receptor 1, pTNFα – plasma tumour necrosis factor α, pIL-6 – plasma interleukin-6, sIP-10 – serum interferon gamma - induced protein-10, pMIP-1α – plasma macrophage inflammatory protein 1α, pMCP-1 – plasma monocyte chemotactic protein 1, pNGAL – plasma neutrophil gelatinase-associated lipocalin, sTNFsr2 – serum tumour necrosis factor soluble receptor 2, pIL-8 – plasma interleukin 8, sIL-1RA – serum interleukin 1 receptor antagonist, sMK – serum midkine

Supplemental Table 20. Ratios of urinary anti-inflammatory/urinary pro-inflammatory cytokines (Day 5)

| **Urinary anti-inflammatory/ urinary pro-inflammatory cytokines** | **Pre-op**  **or**  **post-op** | **Day 5** | | | | |
| --- | --- | --- | --- | --- | --- | --- |
|  |  | **non-CS-AKI** | | **CS-AKI** | | **p value** |
|  |  | **n** | **ratio** | **n** | **ratio** |  |
| uTNFsr1/ uIL-12p40 | post-op | 203 | 1.566 | 17 | 0.930 | 0.029 |
| uTNFsr2/ uIP-10 | post-op | 276 | 0.460 | 21 | 0.372 | 0.041 |
| uTNFsr2/ uIL-12p40 | post-op | 203 | 1.863 | 17 | 1.211 | 0.033 |
| uTNFsr2/ uNGAL | post-op | 285 | 0.052 | 21 | 0.040 | 0.036 |
| uIL-1RA/ uIP-10 | post-op | 276 | 125.720 | 21 | 69.580 | 0.017 |
| uIL-1RA/ uIL-12p40 | post-op | 204 | 480.156 | 17 | 338.779 | 0.019 |
| uIL-1RA/ uNGAL | post-op | 285 | 12.685 | 21 | 7.803 | 0.010 |

pre-op – preoperative, post-op – postoperative, CS-AKI – cardiac surgery acute kidney injury, n – number of patients, uTNFsr1 – urinary tumour necrosis factor soluble receptor 1, uIL-12p40 – urinary interleukin-12 subunit p40, uTNFsr2 – urinary tumour necrosis factor soluble receptor 2, uIP-10 – urinary interferon gamma - induced protein-10, uNGAL – urinary neutrophil gelatinase-associated lipocalin, uIL-1RA – urinary interleukin-1 receptor antagonist

Supplemental Table 21. Ratios of urinary anti-inflammatory/blood pro-inflammatory cytokines (Day 5)

| **Urinary anti-inflammatory/ blood pro-inflammatory cytokines** | **Pre-op**  **or**  **post-op** | **Day 5** | | | | |
| --- | --- | --- | --- | --- | --- | --- |
|  |  | **non-CS-AKI** | | **CS-AKI** | | **p value** |
|  |  | **n** | **ratio** | **n** | **ratio** |  |
| uTNFsr2/ pIL-8 | post-op | 290 | 0.854 | 22 | 0.606 | 0.037 |
| uTNFsr2/ sIL-12p40 | post-op | 287 | 0.029 | 22 | 0.021 | 0.019 |
| uTNFsr2/ pMIP-1α | post-op | 286 | 1.633 | 22 | 1.229 | 0.014 |
| uTNFsr2/ sMK | post-op | 241 | 0.007 | 18 | 0.003 | 0.033 |
| uTNFsr2/ pNGAL | post-op | 290 | 0.008 | 22 | 0.006 | 0.007 |
| uIL-1RA/ pTNFα | post-op | 249 | 704.104 | 21 | 543.804 | 0.024 |
| uIL-1RA/ pIL-6 | pre-op | 271 | 554.184 | 22 | 361.242 | 0.039 |
| uIL-1RA/ pIL-8 | post-op | 291 | 208.970 | 22 | 123.796 | 0.010 |
| uIL-1RA/ sIL-12p40 | pre-op | 294 | 3.537 | 22 | 1.851 | 0.001 |
| uIL-1RA/ sIL-12p40 | post-op | 288 | 6.381 | 22 | 3.938 | 0.002 |
| uIL-1RA/ pMIP-1α | pre-op | 283 | 427.891 | 22 | 343.930 | 0.032 |
| uIL-1RA/ pMIP-1α | post-op | 287 | 384.318 | 22 | 252.012 | <0.001 |
| uIL-1RA/ pMCP-1 | post-op | 291 | 9.029 | 22 | 5.890 | 0.015 |
| uIL-1RA/ sMK | post-op | 242 | 1.766 | 18 | 0.718 | 0.008 |
| uIL-1RA/ pNGAL | pre-op | 288 | 2.616 | 22 | 1.593 | 0.017 |
| uIL-1RA/ pNGAL | post-op | 291 | 1.840 | 22 | 1.110 | <0.001 |

pre-op – preoperative, post-op – postoperative, CS-AKI – cardiac surgery acute kidney injury, n – number of patients, uTNFsr2 – urinary tumour necrosis factor soluble receptor 2, pIL-8 – plasma interleukin 8, sIL-12p40 – serum interleukin 12 subunit p40, pMIP-1α – plasma macrophage inflammatory protein 1α, sMK – serum midkine, pNGAL – plasma neutrophil gelatinase-associated lipocalin, uIL-1RA – urinary interleukin-1 receptor antagonist, pTNFα – plasma tumour necrosis factor α, pIL-6 – plasma interleukin-6, pIL-8 – plasma interleukin 8, pMCP-1 – plasma monocyte chemotactic protein 1

Supplemental Table 22. Blood hypoperfusion biomarkers (Day 5)

| **Blood hypoperfusion biomarkers** | **Pre-op**  **or**  **post-op** | **Day 5** | | | | |
| --- | --- | --- | --- | --- | --- | --- |
|  |  | **non-CS-AKI** | | **CS-AKI** | | **p value** |
|  |  | **n** | **median** | **n** | **median** |  |
| **Serum biomarker** | | | | | | |
| sH-FABP | post-op | 296 | 19.885 | 22 | 38.735 | 0.002 |
| **Plasma biomarker** | | | | | | |
| pVEGF | pre-op | 292 | 30.500 | 22 | 46.190 | <0.001 |

pre-op – preoperative, post-op – postoperative, CS-AKI – cardiac surgery acute kidney injury, n – number of patients, sH-FABP – serum heart-type fatty acid-binding protein, pVEGF – plasma vascular endothelial growth factor

Supplemental Table 23. Ratios of urinary anti-inflammatory/blood hypoperfusion biomarkers (Day 5)

| **Urinary anti-inflammatory/ blood hypoperfusion biomarkers** | **Pre-op**  **or**  **post-op** | **Day 5** | | | | |
| --- | --- | --- | --- | --- | --- | --- |
|  |  | **non-CS-AKI** | | **CS-AKI** | | **p value** |
|  |  | **n** | **ratio** | **n** | **ratio** |  |
| uTNFsr1/ sH-FABP | post-op | 287 | 0.317 | 22 | 0.169 | 0.017 |
| uTNFsr2/ sH-FABP | post-op | 288 | 0.399 | 22 | 0.196 | 0.005 |
| uIL-1RA/ sH-FABP | post-op | 280 | 3.025 | 21 | 2.514 | 0.006 |
| uIL-1RA/ pVEGF | pre-op | 265 | 49.380 | 22 | 32.530 | 0.008 |

pre-op – preoperative, post-op – postoperative, CS-AKI – cardiac surgery acute kidney injury, n – number of patients, uTNFsr1 – urinary tumour necrosis factor soluble receptor 1, sH-FABP – serum heart-type fatty acid-binding protein, uTNFsr2 – urinary tumour necrosis factor soluble receptor 2, uIL-1RA – urinary interleukin-1 receptor antagonist

Supplemental Table 24. Ratios of blood anti-inflammatory/urinary anti-inflammatory cytokines (Day 5)

| **Blood anti-inflammatory/ urinary anti-inflammatory cytokines** | **Pre-op**  **or**  **post-op** | **Day 5** | | | | |
| --- | --- | --- | --- | --- | --- | --- |
|  |  | **non-CS-AKI** | | **CS-AKI** | | **p value** |
|  |  | **n** | **ratio** | **n** | **ratio** |  |
| sTNFsr1/ uTNFsr1 | pre-op | 293 | 0.675 | 22 | 1.009 | 0.050 |
| sTNFsr1/ uTNFsr1 | post-op | 286 | 0.109 | 22 | 0.159 | 0.021 |
| sTNFsr1/ uTNFsr2 | pre-op | 281 | 0.371 | 22 | 0.667 | 0.050 |
| sTNFsr1/ uTNFsr2 | post-op | 287 | 0.088 | 22 | 0.128 | 0.001 |
| sTNFsr1/ uIL-1RA | pre-op | 294 | 0.0002 | 22 | 0.0004 | 0.002 |
| sTNFsr1/ uIL-1RA | post-op | 288 | 0.0004 | 22 | 0.0006 | <0.001 |
| sTNFsr2/ uTNFsr1 | pre-op | 293 | 0.684 | 22 | 1.034 | 0.050 |
| sTNFsr2/ uTNFsr1 | post-op | 286 | 0.121 | 22 | 0.180 | 0.028 |
| sTNFsr2/ uTNFsr2 | pre-op | 281 | 0.431 | 22 | 0.547 | 0.050 |
| sTNFsr2/ uTNFsr2 | post-op | 287 | 0.099 | 22 | 0.135 | 0.003 |
| sTNFsr2/ uIL-1RA | pre-op | 294 | 0.0003 | 22 | 0.0005 | 0.004 |
| sTNFsr2/ uIL-1RA | post-op | 288 | 0.0004 | 22 | 0.0007 | <0.001 |
| sIL-1RA/ uTNFsr1 | post-op | 286 | 68.847 | 22 | 149.826 | 0.014 |
| sIL-1RA/ uTNFsr2 | post-op | 287 | 56.775 | 22 | 120.819 | 0.003 |
| sIL-1RA/ uIL-1RA | post-op | 288 | 0.237 | 22 | 0.653 | <0.001 |

pre-op – preoperative, post-op – postoperative, CS-AKI – cardiac surgery acute kidney injury, n – number of patients, sTNFsr1 – serum tumour necrosis factor soluble receptor 1, uTNFsr1 – urinary tumour necrosis factor soluble receptor 1, uTNFsr2 – urinary tumour necrosis factor soluble receptor 2, uIL-1RA – urinary interleukin-1 receptor antagonist, sTNFsr2 – serum tumour necrosis factor soluble receptor 2, sIL-1RA – serum interleukin-1 receptor antagonist

Supplemental Table 25. Ratios of blood anti-inflammatory/blood pro-inflammatory cytokines (Any day)

| **Blood anti-inflammatory/ blood pro-inflammatory cytokines** | **Pre-op**  **or**  **post-op** | **Any day** | | | | |
| --- | --- | --- | --- | --- | --- | --- |
|  |  | **non-CS-AKI** | | **CS-AKI** | | **p value** |
|  |  | **n** | **ratio** | **n** | **ratio** |  |
| sTNFsr1/ pTNFα | pre-op | 208 | 0.156 | 59 | 0.195 | <0.001 |
| sTNFsr1/ pTNFα | post-op | 211 | 0.264 | 61 | 0.329 | <0.001 |
| sTNFsr1/ pIL-6 | post-op | 250 | 0.006 | 65 | 0.007 | 0.036 |
| sTNFsr1/ pIL-8 | pre-op | 253 | 0.124 | 64 | 0.144 | 0.032 |
| sTNFsr1/ sIP-10 | pre-op | 260 | 0.003 | 64 | 0.004 | 0.013 |
| sTNFsr1/ pMIP-1α | pre-op | 250 | 0.098 | 63 | 0.121 | 0.001 |
| sTNFsr1/ pMCP-1 | pre-op | 253 | 0.003 | 64 | 0.004 | <0.001 |
| sTNFsr1/ pMCP-1 | post-op | 251 | 0.003 | 65 | 0.004 | 0.003 |
| sTNFsr1/ pNGAL | pre-op | 254 | 0.0006 | 64 | 0.0008 | <0.001 |
| sTNFsr2/ pTNFα | pre-op | 208 | 0.150 | 59 | 0.215 | <0.001 |
| sTNFsr2/ pTNFα | post-op | 211 | 0.290 | 61 | 0.368 | <0.001 |
| sTNFsr2/ pIL-6 | post-op | 250 | 0.006 | 65 | 0.009 | 0.005 |
| sTNFsr2/ pIL-8 | pre-op | 253 | 0.115 | 64 | 0.172 | 0.003 |
| sTNFsr2/ pIL-8 | post-op | 251 | 0.081 | 65 | 0.100 | 0.045 |
| sTNFsr2/ sIP-10 | pre-op | 260 | 0.003 | 64 | 0.004 | 0.011 |
| sTNFsr2/ sIP-10 | post-op | 254 | 0.007 | 65 | 0.009 | 0.016 |
| sTNFsr2/ pMIP-1α | pre-op | 250 | 0.100 | 63 | 0.130 | 0.001 |
| sTNFsr2/ pMCP-1 | pre-op | 253 | 0.003 | 64 | 0.004 | <0.001 |
| sTNFsr2/ pMCP-1 | post-op | 251 | 0.004 | 65 | 0.005 | <0.001 |
| sTNFsr2/ sMK | pre-op | 210 | 0.0006 | 58 | 0.0009 | 0.008 |
| sTNFsr2/ pNGAL | pre-op | 254 | 0.0007 | 64 | 0.0009 | <0.001 |
| sIL-1RA/ pTNFα | post-op | 211 | 174.510 | 61 | 276.730 | 0.001 |
| sIL-1RA/ pIL-6 | post-op | 250 | 3.746 | 65 | 5.553 | 0.004 |
| sIL-1RA/ pIL-8 | post-op | 251 | 47.448 | 65 | 64.913 | 0.026 |
| sIL-1RA/ sIP-10 | post-op | 254 | 4.088 | 65 | 6.397 | 0.014 |
| sIL-1RA/ pMCP-1 | post-op | 251 | 2.053 | 65 | 3.186 | 0.001 |
| sIL-1RA/ pNGAL | post op | 252 | 0.446 | 65 | 0.715 | 0.035 |

pre-op – preoperative, post-op – postoperative, CS-AKI – cardiac surgery acute kidney injury, n – number of patients, sTNFsr1 – serum tumour necrosis factor soluble receptor 1, pTNFα – plasma tumour necrosis factor α, pIL-6 – plasma interleukin-6, pIL-8 – plasma interleukin 8, sIP-10 – serum interferon gamma - induced protein-10, pMIP-1α – plasma macrophage inflammatory protein 1α, pMCP-1 – plasma monocyte chemotactic protein 1, pNGAL – plasma neutrophil gelatinase-associated lipocalin, sTNFsr2 – serum tumour necrosis factor soluble receptor 2, sMK – serum midkine, sIL-1RA – serum interleukin 1 receptor antagonist

Supplemental Table 26. Ratios of urinary anti-inflammatory/urinary pro-inflammatory cytokines (Any day)

| **Urinary anti-inflammatory/ urinary pro-inflammatory cytokines** | **Pre-op**  **or**  **post-op** | **Any day** | | | | |
| --- | --- | --- | --- | --- | --- | --- |
|  |  | **non-CS-AKI** | | **CS-AKI** | | **p value** |
|  |  | **n** | **ratio** | **n** | **ratio** |  |
| uTNFsr1/ uIP-10 | post-op | 237 | 0.410 | 63 | 0.314 | 0.048 |
| uTNFsr1/ uNGAL | post-op | 245 | 0.044 | 64 | 0.037 | 0.049 |
| uTNFsr2/ uIP-10 | post-op | 238 | 0.478 | 63 | 0.335 | 0.004 |
| uTNFsr2/ uNGAL | post-op | 246 | 0.053 | 64 | 0.040 | 0.004 |
| uIL-1RA/ uIP-10 | post-op | 238 | 127.555 | 63 | 81.196 | 0.001 |
| uIL-1RA/ uIL-12p40 | post-op | 169 | 512.772 | 54 | 350.832 | 0.001 |
| uIL-1RA/ uNGAL | post-op | 246 | 13.551 | 64 | 8.051 | <0.001 |

pre-op – preoperative, post-op – postoperative, CS-AKI – cardiac surgery acute kidney injury, n – number of patients, uTNFsr1 – urinary tumour necrosis factor soluble receptor 1, uIP-10 – urinary interferon gamma - induced protein-10, uNGAL – urinary neutrophil gelatinase-associated lipocalin, uTNFsr2 – urinary tumour necrosis factor soluble receptor 2, uIL-1RA – urinary interleukin-1 receptor antagonist, uIL-12p40 – urinary interleukin-12 subunit p40

Supplemental Table 27. Ratios of urinary anti-inflammatory/blood pro-inflammatory cytokines (Any day)

| **Urinary anti-inflammatory/ blood pro-inflammatory cytokines** | **Pre-op**  **or**  **post-op** | **Any day** | | | | |
| --- | --- | --- | --- | --- | --- | --- |
|  |  | **non-CS-AKI** | | **CS-AKI** | | **p value** |
|  |  | **n** | **ratio** | **n** | **ratio** |  |
| uTNFsr1/ sIL-12p40 | post-op | 247 | 0.023 | 65 | 0.019 | 0.015 |
| uTNFsr1/ sMK | post-op | 204 | 0.006 | 58 | 0.003 | 0.003 |
| uTNFsr2/ sIL-12p40 | post-op | 248 | 0.030 | 65 | 0.022 | 0.001 |
| uTNFsr2/ pMIP-1α | post-op | 247 | 1.638 | 65 | 1.421 | 0.026 |
| uTNFsr2/ sMK | post-op | 205 | 0.008 | 58 | 0.004 | <0.001 |
| uTNFsr2/ pNGAL | post-op | 251 | 0.008 | 65 | 0.006 | 0.007 |
| uIL-1RA/ pTNFα | post-op | 213 | 725.734 | 61 | 586.286 | 0.002 |
| uIL-1RA/ pIL-6 | pre-op | 233 | 583.157 | 64 | 382.638 | 0.004 |
| uIL-1RA/ pIL-6 | post-op | 251 | 16.614 | 65 | 12.810 | 0.028 |
| uIL-1RA/ pIL-8 | post-op | 252 | 214.216 | 65 | 157.119 | 0.030 |
| uIL-1RA/ sIP-10 | post-op | 249 | 17.685 | 65 | 14.805 | 0.039 |
| uIL-1RA/ sIL-12p40 | pre-op | 256 | 3.588 | 64 | 2.856 | <0.001 |
| uIL-1RA/ sIL-12p40 | post-op | 249 | 7.080 | 65 | 4.197 | <0.001 |
| uIL-1RA/ pMIP-1α | pre-op | 246 | 439.349 | 63 | 367.121 | 0.014 |
| uIL-1RA/ pMIP-1α | post-op | 248 | 421.239 | 65 | 263.916 | <0.001 |
| uIL-1RA/ pMCP-1 | post-op | 252 | 9.228 | 65 | 7.067 | <0.001 |
| uIL-1RA/ sMK | post-op | 206 | 1.917 | 58 | 0.789 | <0.001 |
| uIL-1RA/ pNGAL | pre-op | 250 | 2.634 | 64 | 2.057 | 0.011 |
| uIL-1RA/ pNGAL | post-op | 252 | 1.951 | 65 | 1.214 | <0.001 |

pre-op – preoperative, post-op – postoperative, CS-AKI – cardiac surgery acute kidney injury, n – number of patients, uTNFsr1 – urinary tumour necrosis factor soluble receptor 1, sIL-12p40 – serum interleukin-12 subunit p40, sMK – serum midkine, uTNFsr2 – urinary tumour necrosis factor soluble receptor 2, pMIP-1α – plasma macrophage inflammatory protein 1α, pNGAL – plasma neutrophil gelatinase-associated lipocalin, uIL-1RA – urinary interleukin-1 receptor antagonist, pTNFα – plasma tumour necrosis factor α, pIL-6 – plasma interleukin-6, pIL-8 – plasma interleukin 8, sIP-10 – serum interferon gamma - induced protein-10, pMCP-1 – plasma monocyte chemotactic protein 1

Supplemental Table 28. Blood hypoperfusion biomarkers (Any day)

| **Blood hypoperfusion biomarkers** | **Pre-op**  **or**  **post-op** | **Any day** | | | | |
| --- | --- | --- | --- | --- | --- | --- |
|  |  | **non-CS-AKI** | | **CS-AKI** | | **p value** |
|  |  | **n** | **median** | **n** | **median** |  |
| **Serum biomarker** | | | | | | |
| sH-FABP | pre-op | 259 | 4.810 | 63 | 6.530 | 0.001 |
| sH-FABP | post-op | 257 | 17.160 | 65 | 43.220 | <0.001 |
| **Plasma biomarker** | | | | | | |
| pVEGF | pre-op | 254 | 30.000 | 64 | 35.000 | 0.002 |

pre-op – preoperative, post-op – postoperative, CS-AKI – cardiac surgery acute kidney injury, n – number of patients, sH-FABP – serum heart-type fatty acid-binding protein, pVEGF – plasma vascular endothelial growth factor

Supplemental Table 29. Ratios of urinary anti-inflammatory/blood hypoperfusion biomarkers (Any day)

| **Urinary anti-inflammatory/ blood hypoperfusion biomarkers** | **Pre-op**  **or**  **post-op** | **Any day** | | | | |
| --- | --- | --- | --- | --- | --- | --- |
|  |  | **non-CS-AKI** | | **CS-AKI** | | **p value** |
|  |  | **n** | **ratio** | **n** | **ratio** |  |
| uTNFsr1/ sH-FABP | post-op | 248 | 0.329 | 65 | 0.168 | <0.001 |
| uTNFsr2/ sH-FABP | post-op | 249 | 0.439 | 65 | 0.196 | <0.001 |
| uIL-1RA/ sH-FABP | pre-op | 242 | 4.454 | 62 | 3.923 | 0.023 |
| uIL-1RA/ sH-FABP | post-op | 241 | 3.146 | 64 | 2.547 | <0.001 |

pre-op – preoperative, post-op – postoperative, CS-AKI – cardiac surgery acute kidney injury, n – number of patients, uTNFsr1 – urinary tumour necrosis factor soluble receptor 1, sH-FABP – serum heart-type fatty acid-binding protein, uTNFsr2 – urinary tumour necrosis factor soluble receptor 2, uIL-1RA – urinary interleukin-1 receptor antagonist

Supplemental Table 30. Ratios of blood anti-inflammatory/urinary anti-inflammatory cytokines (Any day)

| **Blood anti-inflammatory/ urinary anti-inflammatory cytokines** | **Pre-op**  **or**  **post-op** | **Any day** | | | | |
| --- | --- | --- | --- | --- | --- | --- |
|  |  | **non-CS-AKI** | | **CS-AKI** | | **p value** |
|  |  | **n** | **ratio** | **n** | **ratio** |  |
| sTNFsr1/ uTNFsr1 | post-op | 247 | 0.108 | 65 | 0.118 | 0.028 |
| sTNFsr1/ uTNFsr2 | post-op | 248 | 0.087 | 65 | 0.117 | 0.002 |
| sTNFsr1/ uIL-1RA | pre-op | 256 | 0.0002 | 64 | 0.0003 | <0.001 |
| sTNFsr1/ uIL-1RA | post-op | 249 | 0.0004 | 65 | 0.0006 | <0.001 |
| sTNFsr2/ uTNFsr1 | pre-op | 255 | 0.676 | 64 | 0.997 | 0.044 |
| sTNFsr2/ uTNFsr1 | post-op | 247 | 0.117 | 65 | 0.157 | 0.004 |
| sTNFsr2/ uTNFsr2 | post-op | 248 | 0.096 | 65 | 0.136 | <0.001 |
| sTNFsr2/ uIL-1RA | pre-op | 256 | 0.0002 | 64 | 0.0004 | <0.001 |
| sTNFsr2/ uIL-1RA | post-op | 249 | 0.0004 | 65 | 0.0007 | <0.001 |
| sIL-1RA/ uTNFsr1 | post-op | 247 | 67.434 | 65 | 93.093 | 0.016 |
| sIL-1RA/ uTNFsr2 | post-op | 248 | 55.121 | 65 | 98.477 | 0.002 |
| sIL-1RA/ uIL-1RA | pre-op | 256 | 0.045 | 64 | 0.054 | 0.015 |
| sIL-1RA/ uIL-1RA | post-op | 249 | 0.224 | 65 | 0.468 | <0.001 |

pre-op – preoperative, post-op – postoperative, CS-AKI – cardiac surgery acute kidney injury, n – number of patients, sTNFsr1 – serum tumour necrosis factor soluble receptor 1, uTNFsr1 – urinary tumour necrosis factor soluble receptor 1, uTNFsr2 – urinary tumour necrosis factor soluble receptor 2, uIL-1RA – urinary interleukin-1 receptor antagonist, sTNFsr2 – serum tumour necrosis factor soluble receptor 2, sIL-1RA – serum interleukin-1 receptor antagonist
